# Effective interactions and phase behavior of protein solutions in the presence of hexamine cobalt(III) chloride

**DOI:** 10.1140/epje/s10189-023-00376-6

**Published:** 2023-12-05

**Authors:** Maximilian D. Senft, Ralph Maier, Anusha Hiremath, Fajun Zhang, Frank Schreiber

**Affiliations:** Institut für Angewandte Physik, Universität Tübingen, Auf der Morgenstelle 10, 72076 Tübingen, Germany

## Abstract

It is well established that deoxyribonucleic acid (DNA) and ribonucleic acid (RNA) exhibit a reentrant condensation (RC) phase behavior in the presence of the trivalent hexamine cobalt(III) cations (Hac) which can be important for their packing and folding. A similar behavior can be observed for negatively charged globular proteins in the presence of trivalent metal cations, such as Y^3+^ or La^3+^. This phase behavior is mainly driven by charge inversion upon an increasing salt concentration for a fixed protein concentration (*c*_p_). However, as Hac exhibits structural differences compared to other multivalent metal cations, with six ammonia ligands (NH_3_) covalently bonded to the central cobalt atom, it is not clear that Hac can induce a similar phase behavior for proteins. In this work, we systematically investigate whether negatively charged globular proteins β-lactoglobulin (BLG), bovine serum albumin (BSA), human serum albumin (HSA) and ovalbumin (OVA) feature Hac-induced RC. Effective protein–protein interactions were investigated by small-angle X-ray scattering. The reduced second virial coefficient (B_2_/B_2_^HS^) was obtained as a function of salt concentration. The virial coefficient analysis performed confirms the reentrant interaction (RI) behavior for BLG without actually inducing RC, given the insufficient strengths of the interactions for the latter to occur. In contrast, the strength of attraction for BSA, HSA and OVA are too weak to show RC. Model free analysis of the inverse intensity $1/I\left( {q \to 0} \right)$ also supports this finding. Looking at different *q*-range by employing static (SLS) and dynamic light scattering experiments, the presence of RI behavior can be confirmed. The results are further discussed in view of metal cation binding sites in nucleic acids (DNA and RNA), where Hac induced RC phase behavior.

**Graphical abstract:**

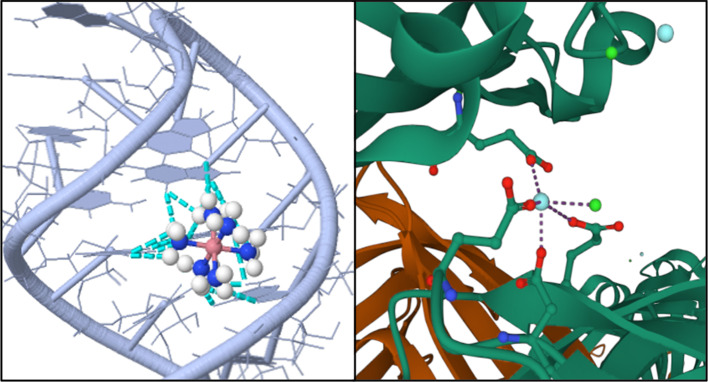

**Supplementary Information:**

The online version contains supplementary material available at 10.1140/epje/s10189-023-00376-6.

## Introduction

Effective protein–protein interactions determine the phase behavior of proteins in aqueous solutions, including liquid–liquid phase separation (LLPS) and crystallization. Understanding as well as predicting the phase behavior of proteins is therefore not only beneficial for X-ray structural analysis of protein crystals, but also for a more profound understanding of diseases related to the aggregation of proteins [1–3]. Furthermore, the occurrence of metastable LLPS in protein solutions represents an important mechanism for biological structure formation [1, 2, 4–11]. Diseases such as eye cataract, [8, 9] lateral sclerosis, [6, 7] sickle cell anemia, [5, 11] Alzheimer’s disease in line with amyloidosis [4, 6] are related to unwanted protein fiber formation, crystallization, or aggregation.

In aqueous solution, the effective interactions of proteins turn out to be rather complex, as various environmental parameters [3, 10, 12, 13] such as the protein concentration, concentration and valence of the salt ions, temperature and pH-value strongly influence their interactions [14]. Moreover, protein–protein interactions are determined by the protein's surface charge pattern or by a modulation of hydrophilic and hydrophobic interactions. In combination with Coulomb interactions, hydrogen bonding, and specific or non-specific salt bridging, this interplay of multiple interactions results in a rich phase behavior of aqueous protein solutions. Further studies, focusing on the modeling of liquid–liquid phase transitions in protein- and colloid- systems, pointed out the relevance of short-ranged attractive forces [15–19].

Reentrant condensation (RC) is an intriguing phenomenon which occurs in various acidic, globular proteins, given the presence of multivalent metal salts such as YCl_3_ [14, 20–23]. For a fixed protein concentration (*c*_p_) combined with a salt concentration below the value of c*, or above a second value c**, with c* < c**, the protein solution appears translucent. If the salt concentration is between c* and c**, protein condensation occurs, which may include LLPS, aggregation, and protein crystallization [12, 20, 21, 24, 25]. Responsible for the reentrant condensation behavior are both the cation-mediated inversion of the protein charge and the intermittent cation-mediated attraction [26]. Charge reversal and effective attraction induced by multivalent metal ions have been further investigated not only in theoretical studies but also in experiments and simulations [26–29]. In order to fundamentally understand the origin of the macroscopic phase behavior and the involved interactions, the underlying forces need to be investigated [20].

Deoxyribonucleic acid (DNA) has been known to condense in aqueous solution in the presence of multivalent cations, such as hexamine cobalt(III) (Hac), for a long time [30]. Due to advancing research in the field of gene therapy, interest in the phenomenon of DNA condensation with multivalent cations increased. The goal of this research field is to develop efficient ways of gene transfer, which requires a simple, effective, and yet reversible method without damaging the DNA in the process [31].

The relatively inert trivalent cation hexamine cobalt(III) features six tightly bound amino groups which inhibits the option of direct coordinate ion bonds between the cobalt and polar groups of the DNA helix, [30] therefore fulfilling the above required conditions [31]. In order to enable DNA condensation, approximately three out of four negative charges have to be neutralized by a bound cation [32]. In other words, approximately 90% of the total charges exhibited by DNA have to be neutralized to allow for condensation [33, 34]. DNA neutralization enables and facilitates the compression of DNA as it occurs in the genomes of cells or viruses as well as the deformation of DNA mediated through proteins [32]. When trivalent ions are present in a solution with DNA, the DNA also undergoes RC. It should be emphasized that the driving force for the condensation of DNA is the electrostatic interaction between the cations and the DNA. Furthermore, this leads to attractive, correlated counter-ion fluctuations. Thereby, the counterions also screen Coulombic repulsions between DNA phosphates before the condensation takes place [32, 34]. Apart from DNA condensation, investigations were also performed on Ribonucleic acid (RNA) [35–37].

Moreover, Hac proved to be of use in other biological research areas. Hac has been successfully deployed to inhibit an RNA polymerase of West Nile virus in order to investigate the role of magnesium ions, necessary for the catalysis carried out by this RNA polymerase [38]. Furthermore, Hac was used in transformations with *Escherichia coli* to either change the confirmation of the DNA or serve as a counterpart of vitamin B_12_. Here, it either interferes with the constituents of the vitamin or disrupts its transport system [39]. In addition, Hac has also been proven to possess anti-bacterial and anti-viral effects [40]. The element cobalt (Co) is a standard component of metal alloys [41] which are, among others, used for biomedical implants (e.g., hip prothesis). Expedient wear in combination with corrosion of implants leads to in vivo release of metal ions [42, 43]. This may support unwanted protein aggregation, triggering associated diseases such as lateral sclerosis or Alzheimer’s [44, 45], and may as well trigger metal contact hypersensitivity [45].

When comparing proteins with DNA or conventional colloid suspensions, which are governed by identical charges, it turns out that on the surface positive as well as negative charges are present. These charges are arranged in a rather complex, unevenly distributed pattern, resulting in differences regarding the interactions. Moreover, a DNA molecule can be approximated with a thin extended rod-like shape, whereas the proteins examined here feature a globular geometry. In addition, the complex surface charge pattern, together with other kinds of interactions, like hydrophobic interactions or hydration, increase the complexity of the phase behavior for proteins in solution [3, 13, 14]. This work conducts a systematic study of the effective interactions and phase behavior of the proteins BLG, BSA, HSA, and OVA in aqueous solution in the presence of the trivalent salt hexamine cobalt(III) chloride. All mentioned proteins already featured RC behavior induced by several trivalent metal salts [46].

We aim to explore how different concentrations of Hac can tune the effective interactions in these protein solutions and if it is sufficient to induce RC phase behavior. For that, the effective protein–protein interactions were evaluated based on the analysis of the reduced second virial coefficient from Small-angle X-ray scattering (SAXS) measurements. In addition, static light scattering (SLS) and dynamic light scattering (DLS) experiments were carried out to explore a different *q*-range and to investigate diffusive dynamics. The results are further discussed in view of metal cation binding sites in nucleic acids (DNA and RNA), where Hac induced RC phase behavior.

## Experimental section

### Materials and sample preparation

The proteins BLG from bovine milk (product no. L3908, purity of ≥ 90%), BSA (product no. A3733, purity of ≥ 98%), HSA (product no. A9511, purity of ≥ 97%) and OVA from chicken egg white (product no. A5503, purity of ≥ 98%) were purchased from Merck and used in the experiments without additional purification. Fundamental biophysiochemical properties of the proteins are summarized in Table 1. Note that the purchased protein β-lactoglobulin (BLG) is a mixture of the two genetic variants A and B, which differ only at two locations within their primary sequence [47]. Moreover, given physiological conditions, BLG features predominantly a dimer configuration [48, 49].

**Table 1:** Biophysiochemical properties of BLG, BSA, HSA, and OVA with respective reference

Parameters	BLG	BSA	HSA	OVA
# Amino acids	162 [47]	583 [50]	585 [51]	385 [52]
Molecular weight (kDa)	37 [53]	66.4 [54]	66.44 [51]	45 [55]
pI	5.2 [48]	4.6 [46]	4.7 [51]	4.54 [56]
Charge (pH 7) (e)	− 10 [57]	− 11 [46]	− 9 [46]	− 11 [58]
Specific volume (ml/g)	0.750 [59]	0.735 [59]	0.754 [60]	0.745 [61]
Extinction coefficient (ml mg^−1^ cm^−1^)	0.961[62]	0.667[62]	0.531[62]	0.700[62]

Protein stock solutions were obtained by mixing the required amount of the respective protein stock with deionized and degassed Millipore water (conductivity of 18.2 MΩ cm). The concentration of the protein stock solution was determined by use of a Cary 50 UV–Vis spectrophotometer (Varian Technologies) with the appropriate extinction coefficients (see Table 1) and the Cary WinUV operating software. The absorbance was measured at 280 nm. The protein solutions were stored in appropriate parafilm-sealed containers to avoid de novo solution of gasses and placed in the fridge at 4 °C [63]. In order to avoid unwanted bacterial or fungi growth, the protein solutions were used for a maximum of three weeks [64].

Hexamine cobalt(III) chloride ([Co(NH_3_)_6_]Cl_3_) powder was purchased from Merck (H7891, for use in transformations, X-ray crystallography), dissolved in deionized and degassed Millipore water and used in the carried out experiments without further purification. This multivalent salt features a molecular weight of 264.48 g/mol and a density of 1.71 g/ml [65]. Systematic deviations arising from variations in protein batches, preparative inaccuracies, and fluctuations in protein and salt stock solutions cannot be ruled out.

### Small-angle X-ray scattering

Small-Angle X-ray Scattering (SAXS) experiments were conducted at the P12 beamline of the EMBL, DESY (Hamburg, Germany) [66]. The system employs a highly focused X-ray beam ($120 \times 200 \,{\mu m})$ with an energy of 10 keV, which corresponds to a wavelength of ($= 1.24$ Å). The sample-to-detector distance was set to 3.1 m. The employed 2 M Pilatus (Dectris) detector covered a *q* range of 0.002–0.45 Å^−1^ [66]. The samples were exchanged by the use of a flow cell. For each sample, 40 exposures of 0.04 s were averaged. Additional SAXS data was collected using the laboratory SAXS instrument Xeuss 2.0 (Xenocs, Grenoble, France) employing a GeniX 3D microfocus X-ray tube consisting of a copper anode, using an X-ray energy of 8.05 keV which corresponds to a wavelength of 1.54 Å. With a sample-to-detector distance of 1666 mm, the employed Pilatus 300 K (Dectris) detector covered a *q* range of 0.0076 Å^−1^ up to 0.344 Å^−1^. The protein solutions were measured in quartz capillaries with a diameter of 1.5–2.0 mm (WJM-Glas Müller GmbH, Berlin, Germany). The acquisition time for each measurement was set to two hours. Each sample preparation was carried out right before the measurements.

The 2D data obtained was azimuthally averaged to yield intensity profiles. Subsequently, the solvent background was measured, treated in a similar manner and finally subtracted from the intensity profiles. Afterward, the background-corrected data was fitted by use of the ellipsoidal sticky hard sphere potential (SHS), originating from Baxter [67], as implemented by the National Institute of Standards and Technology provided add-on for IGOR PRO 6.37 and 9 [68]. The data was analyzed utilizing the same method explained in the references [69, 70]. For a spherical particle of radius *R,* the SHS is defined as follows:

1 $$ \beta U\left( r \right) = \left\{ {\begin{array}{*{20}l} \infty \hfill & {r < \sigma = 2R} \hfill \\ { - \beta u_{0} = \ln \left( {\frac{12\tau \Delta }{{\sigma + \Delta }}} \right)} \hfill & {\sigma < r < \sigma + \Delta } \hfill \\ 0 \hfill & {r > \sigma + \Delta } \hfill \\ \end{array} } \right. $$

here *β* represents *1/k*_*B*_*T*, *τ* represents the stickiness parameter and *Δ* denotes the width of the square well. The diameter of the hard sphere is denoted by *σ*, and *r* denotes the particle spacing. In order to determine the structure factor, a perturbative solution of the Percus–Yevick closure relation was used [67, 71].

In the limit $\Delta \to 0$, the reduced second virial coefficient can be calculated based on

2 $$ \mathop {\lim }\limits_{\Delta \to 0} \frac{{B_{2} }}{{B_{2}^{HS} }} = 1 - \frac{1}{4\tau } $$

As shown in Eq. (2), the reduced second virial coefficient is obtained by dividing the second virial coefficient (*B*_2_) by the second virial coefficient for hard spheres (*B*_2_^*HS*^) of radius *R* given by $B_{2}^{HS} = 16\pi R^{3} {/}3$. Simulations and theories have resulted in a universal *B*_*2*_*/B*_*2*_^*HS*^ value of $\approx - 1.56$ for the liquid–gas transition in a number of different systems, provided the application of the Percus–Yevick closure relation [19, 22, 72].

Moreover, other potentials such as Screened Coulombic (SC), Two Yukawa (2Y) and hard spheres (HS) were used to fit the SAXS data for different protein systems and different salt concentrations. A detailed description of these potentials can be found in the Supporting Information.

Besides the volume fraction, the axes of the ellipsoids (*R*_*a*_ and *R*_*b*_) were fixed to *R*_*a*_ 37.6–39.0 Å, *R*_*b*_ 19.5–20.0 Å for BLG, *R*_*a*_ 17.0 Å and *R*_*b*_ 42.0–44.4 Å for BSA. Detailed information on HSA, and OVA, can be found in Table S3 in the Supporting Information. Moreover, the scattering length density (SLD) of the proteins was set to $7.33 \times 10^{ - 7}$ [Å^−2^] for BLG and to $7.32 \times 10^{ - 7}$ [Å^−2^] for BSA, HSA and OVA respectively. The background was set to appropriate values for each curve individually. In order to prevent artificial coupling between the well width *Δ* and the stickiness parameter *τ*, *Δ* was kept at $0.01 \sigma$ for all fitted data. Further details on the fitting process and selected potentials for fitting of OVA and HSA SAXS-datasets are shown in the Supporting Information. We note that *B*_2_ is a simplified way of quantifying the interactions, inter alia, due to the angular average of non-spherical proteins.

### Static and dynamic light scattering

Further structural and dynamical information was obtained by dynamic light scattering (DLS) experiments, which were conducted on a pure Hac solution (50 mM), a pure BSA (20 mg/ml) solution and several BSA (20 mg/ml) solutions containing increasing concentrations of Hac (1–50 mM) [73]. Prior to the measurements, all samples were filtered using syringe filters (Whatman Puradisc 13; Global Life Sciences Solutions Operations Uk Ltd.) with a 0.45 µm pore size. In this work, an ALV/CGS-3 goniometer with an ALV/LSE-5004 digital correlator (ALV-GmbH, Langen, Germany) operated by the corresponding ALV-Correlator software V 3.0, was used. This instrument utilizes a HeNe-Laser with a wavelength of $\lambda = 6328$ Å as a light source. Quartz glass cuvettes (Pyrex; Corning, Ny, USA) were cleaned with acetone, and after the evaporation of the acetone, filled with the sample solution and subsequently measured.

A calibration to absolute scattering intensities was carried out by the use of a toluene measurement, as standard. Using the Rayleigh ratio $R_{\theta }$, the scattering intensity was calibrated using toluene as a standard [74].

The intensity $\left( {R_{\theta } {/}Kc} \right)$, with $K$ denoting the scattering contrast (Eq. 3) and $c$ denoting the concentration, can be equated to the inverse of the intensity $\left( {Kc{/}R_{\theta } } \right)$ due to the relation of $R\theta \sim I_{{{\text{sample}}}} - I_{{{\text{solvent}}}}$. Additionally, the measured intensity depends on $P\left( q \right)$ of the sample but given the case of small particles $\left( {r < \lambda /20} \right)$, $P\left( q \right)$ can be assumed to be unity within the observed *q*-range, and therefore the scattering is independent from the scattering angle $2\theta$.[74] Based on the aforementioned, it is sufficient to perform the measurements only with one angle. In this work, angles between 70° and 90° were used [74].

3 $$ K = \frac{{4 \pi^{2} n^{2} \left( {dn/dK} \right)^{2} }}{{N_{A } \lambda^{4} }} $$

Changes in the scattering signal over time are of interest in terms of dynamic information. This can be quantified by the use of, the auto correlation function ${\text{g}}_{2} \left( {\text{t}} \right)$ [74–76].

4 $$ g_{2} \left( t \right) = \frac{{\left\langle {I\left( {t_{0} } \right)I\left( {t_{0} - t} \right)} \right\rangle }}{{\left\langle {I\left( {t_{0} } \right)} \right\rangle^{2} }} $$

here $\left\langle \ldots \right\rangle$ denotes a time average, the term $I\left( {t_{0} } \right)$ denotes the scattered intensity at the time $t_{0}$ while $t$ denotes the time difference of the correlator. The characteristic relaxation time *τ*, is obtained by use of the normalized $g_{2} - 1$ Eq. 5, shown below [77].

5 $$ g_{2} \left( t \right) - 1 = \left\{ {A_{1} \exp \left[ { - 2\left( {t / \tau_{1} } \right)} \right] + A_{2} \exp \left[ { - 2\left( {t / \tau_{2} } \right)} \right]} \right\} $$

Due to the presence of two-component dynamics, the fast and slow components are denoted by subscript 1 and 2 respectively. This double exponential fit function (Eq. 5) was used to approximate the auto-correlation function (Eq. 4). All datasets were fitted utilizing Eq. 5 to achieve consistency. The fits were carried out by use of MATLAB.

## Results and discussion

### Visual inspection of the phase behavior

**Fig. 1:**
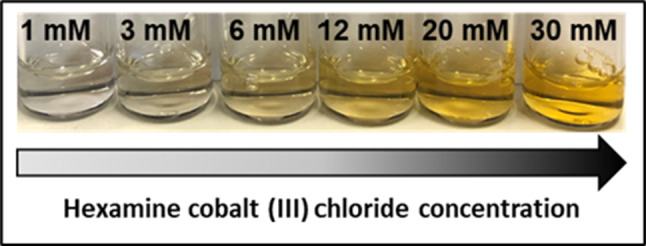
Photograph of BLG with a concentration of 80 mg/ml admixed with increasing Hac concentrations (1–30 mM, left to right). Due to the orange color of the dissolved salt Hac, an increasing yellow tint can be seen at higher salt concentrations. No aggregation is to be seen

First, the phase behavior of various globular proteins was investigated in the presence of Hac at room temperature (21 ± 2 °C). The protein concentration was varied between 5 and 100 mg/ml, while the salt concentration was varied between 1 and 50 mM. All examined proteins showed neither crystal growth nor LLPS or aggregation. This is exemplified for 80 mg/ml BLG in Fig. 1. Owing to the orange color of the dissolved salt, a stronger yellow tint can be seen at higher salt concentrations. Apart from the yellow tint, no opacity or condensates become apparent [14, 20, 21, 46, 70, 78–80]. A similar assessment for HSA and OVA can be found in the Supporting Information.

**Fig. 2:**
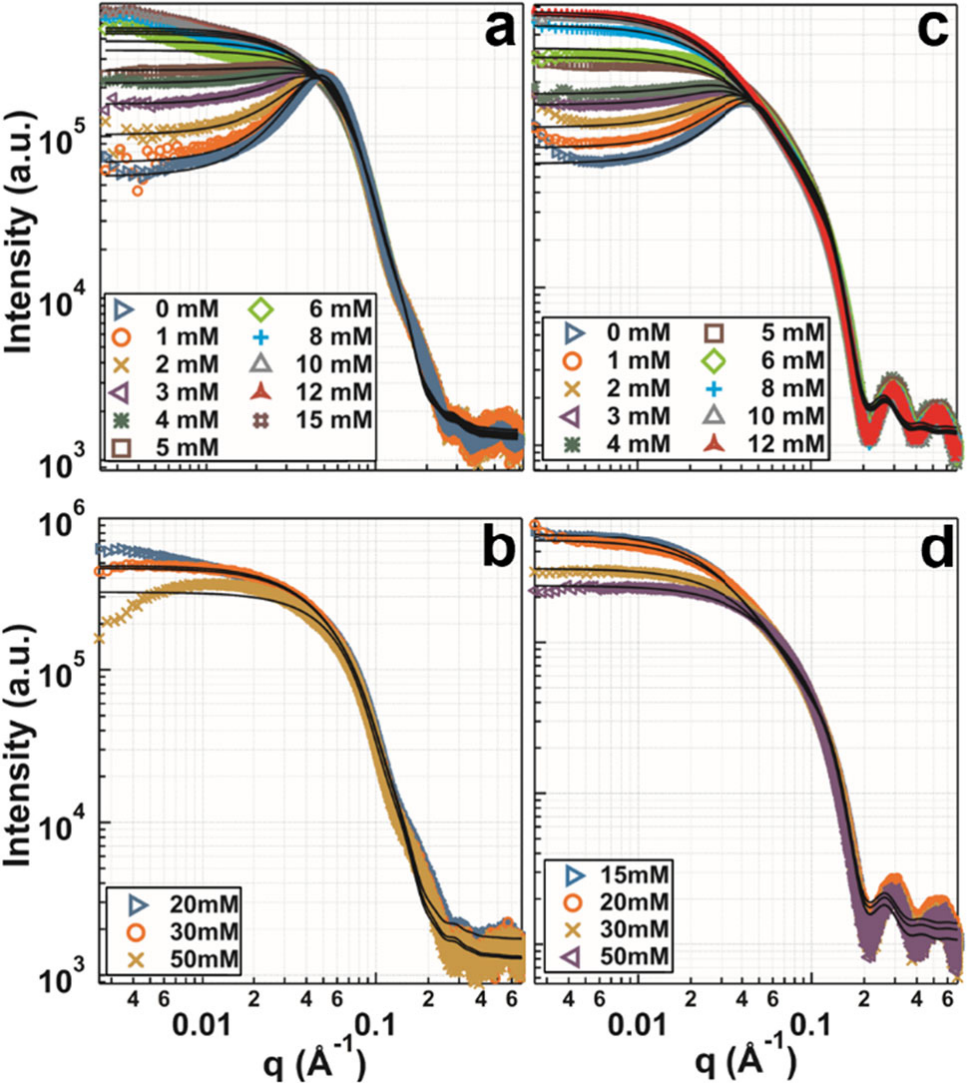
**a**, **b** SAXS data with model fits (solid lines) for samples in H_2_O containing 80 mg/ml BSA with increasing salt concentrations (0–50 mM). **c**, **d** SAXS data with model fits for samples in H_2_O containing 80 mg/ml BLG with increasing salt concentrations (0–50 mM). The scattering intensity at low q increases with increasing salt concentration **a** and **c** and decreases in **b** and **d**. In **a**, the SC potential was used for 0–4 mM salt. The other conditions (5–15 mM) were fitted by use of an SHS model. In **c**, conditions with 0–4 mM salt were fitted using a 2Y potential the other conditions (5–12 mM) were fitted using an SHS model. The dashed lines are guides to the eye. Further detailed information on SAXS data analysis is provided in the Supporting Information

To gain further insight, the effective protein–protein interactions of the protein salt solutions were investigated via systematic SAXS measurements. Figure 2 shows representative SAXS measurements for 80 mg/ml BSA and 80 mg/ml BLG with increasing Hac concentrations in H_2_O (data for OVA and HSA can be found in Figs. S1, S2 in the Supporting Information).

In both protein-salt systems, at low salt concentrations, the net negative charges of the protein molecules dominate the effective protein–protein interactions, as illustrated by a correlation peak at $q = 0.06$ Å^−1^. An increase in the salt concentration results in an increase in low-*q* intensity, which indicates reducing repulsion. Concurrently, the correlation peak fades. As the salt concentration is further increased to 6 mM (for BLG) and 15 mM (for BSA), respectively, attractive interactions dominate the protein-salt system. The highest attractive strength is visible at 15 mM salt for BSA and at 12 mM salt for BLG. At higher salt concentrations (20–50 mM for BSA and 15–50 mM for BLG), the attractive strength reduces, as indicated by the decreasing low-*q* intensities (Figs. 2a and b).

This pattern of increasing and subsequent decreasing low *q* intensities as a function of increasing salt concentration resembles to some extent RC phase behavior and is labeled reentrant interaction (RI) (data for HSA and OVA can be found in the Supporting Information). Qualitatively, from the provided SAXS profiles, it can be deduced that the above characterized behavior is more pronounced for BLG than it is for the other proteins [24, 69, 70, 80]. Previous studies showed that RC is a common phase behavior for BLG, BSA, HSA, and OVA in the presence of either YCl_3_, LaCl_3_, FeCl_3_ or AlCl_3_ [46]. Investigations of RC by means of SAXS have shown that RC is associated with an increasing, followed by a decreasing, intensity at low *q* values, given the prerequisite of a constant protein concentration combined with a continuously increasing salt concentration [69, 70]. Additional data sets for 80 mg/ml OVA and HSA, exhibiting a similar but less pronounced behavior are provided in the Supporting Information (Figs. S1, S2).

### SAXS characterization of the effective interactions

**Fig. 3:**
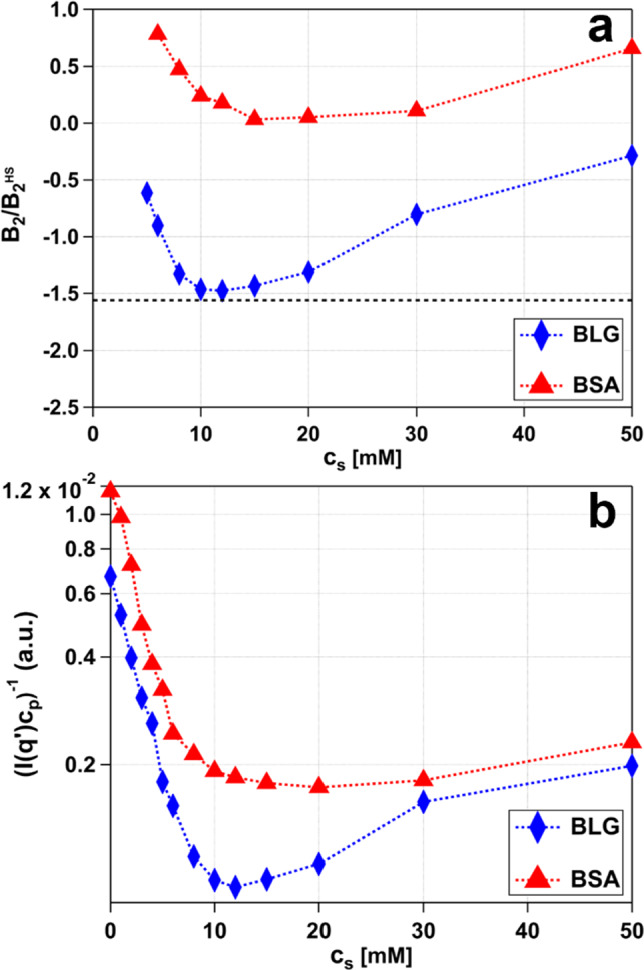
**a** Reduced second virial coefficients and **b** $1/I\left( {q \to 0} \right)$ behavior of the BLG and BSA samples with *c*_p_ 80 mg/ml and varying c_s_ presented in Fig. 2. In **a**, the black dashed line at $- \,1.56$ indicates the suggested and theoretically determined limits of the critical point for the gas–liquid transition (detailed information is provided in the text) [22]. The respective error values of the fits are smaller than the markers used for illustration and are, therefore, not plotted for clarity. **b** The inverse intensities are evaluated at $q^{\prime } = 0.03$ Å^−1^, subsequently averaged, and normalized to the molecular weight of the protein monomer. The respective error values are smaller than the markers used for illustration and are, therefore, not plotted for clarity. The dashed lines are guides to the eye. In the Supporting information, Figs. S3, S4, and S5, show a similar evaluation for the two other proteins OVA and HSA

In order to analyze and quantify the effective protein–protein interactions, models employing an ellipsoidal form factor paired with different interaction potentials were fitted to the SAXS data. Figure 2a displays SC and SHS model fits for BSA and 2Y followed by SHS model fits for BLG (Fig. 2c) with continuously increasing Hac concentrations. Additional fitted SAXS data for the proteins OVA and HSA are included in the Supporting Information. SC interaction potential fits (BSA) and 2Y interaction potential fits (BLG) were confined to low salt concentrations (0–5 mM for BSA and 0–4 mM for BLG). For these conditions, the electrostatic repulsions prevail, owing to net negative surface charges of the respective dissolved proteins. In order to describe the attractive potential arising for higher salt concentrations, the SHS combined with an ellipsoidal form factor was used. In agreement with Eq. (2), the reduced second virial coefficient (*B*_*2*_*/B*_*2*_^*HS*^) was determined. The results of *B*_*2*_/*B*_*2*_^*HS*^ for BLG and BSA are summarized in Fig. 3a as a function of the salt concentration *c*_*s*_. Values below $B_{2} {/}B_{2}^{HS} < 0$ represent net attraction, whereas in the opposite case $B_{2} {/}B_{2}^{HS} > 0$, net repulsion prevails.

As to be seen, the curve for BLG is located below the one for BSA, implying higher attractive strengths. BLG features a local minimum around 12 mM with a corresponding *B*_*2*_*/B*_*2*_^*HS*^ value of $- 1.48$, while BSA features a minimum at 15 mM with a corresponding *B*_*2*_*/B*_*2*_^*HS*^ value of $0.033$. Hence, it can be deduced that BSA remains within the neutral range at 15 mM Hac, while BLG exhibits a more pronounced attraction. Moreover, these two minima hardly touch the universally predicted *B*_*2*_*/B*_*2*_^*HS*^ values of $- 1.56$ [22] at the critical point, marking the theoretical limits for LLPS. Consequently, a phase transition (with LLPS) is absent. Initially, the values for *B*_*2*_*/B*_*2*_^*HS*^ decrease rapidly until the minimum is reached. Beyond the minimum, the *B*_*2*_*/B*_*2*_^*HS*^ values increase again, but with a less steep slope. This effect may be caused by screening effects of the ${\text{Cl}}^{ - }$ counterions. Increasing the salt concentration goes along with increasing concentrations of the counterion, which effectively screens the proteins surface charge. This hypothesis is supported by similar results for divalent cations, which show comparable behavior of the interaction potential as a function of cation concentration [28, 29] A similar evaluation was carried out for the two proteins OVA and HSA. The data can be found in the Supporting Information Figure S3. In addition to the above-described model-based analysis (Fig. 3a), the scattering at low *q* values (close to 0), namely $1/ I\left( {q \to 0} \right)$, was analyzed as well (Fig. 3b). This model-free approach can be applied to repulsive conditions without constraints, unlike the SHS analysis, therefore, allowing to investigate low salt concentrations, too. Importantly, this approach is connected to the reduced second virial coefficient via the following relation [69]:

6 $$ \frac{1}{{I\left( {q \to 0} \right)}} \propto \frac{1}{{S\left( {q \to 0} \right)}} = 1 + 2B_{2} \rho + \cdots $$

hence the value of the structure factor near the origin can be expressed by Eq. 6 which connects the inverse intensity value near the origin to the second virial coefficient [1, 75, 81]. Given the absence of LLPS, the protein concentration is proportional to the protein number density $\left( {c_{p} \propto \rho } \right)$ so the inverse intensity is proportional to the reduced second virial coefficient $\left( {1 /I \left( {q \to 0} \right) \propto B_{2} {/ }B_{2}^{HS} } \right)$ in this approximation. Therefore, the inverse intensity $\left( {1/ I\left( {q \to 0} \right)} \right)$ can be considered as well to describe the effective protein–protein interactions. Moreover, the inverse intensity $\left( {1/ I\left( {q \to 0} \right)} \right)$ was normalized to the respective molar protein concentration in [Mol/l].

Overall, the inverse intensity (Fig. 3b) follows the trend as indicated by the B_2_/B_2_^HS^ analysis (Fig. 3a) for BLG and BSA respectively. The corresponding error bars of the applied fits are smaller than the symbols used for illustration and are therefore not plotted for clarity. Generally, we assume an uncertainty of 10%, resulting from sample preparation and data collection. In the Supporting Information Figs. S4 and S5, show a similar evaluation for the proteins OVA and HSA.

**Fig. 4:**
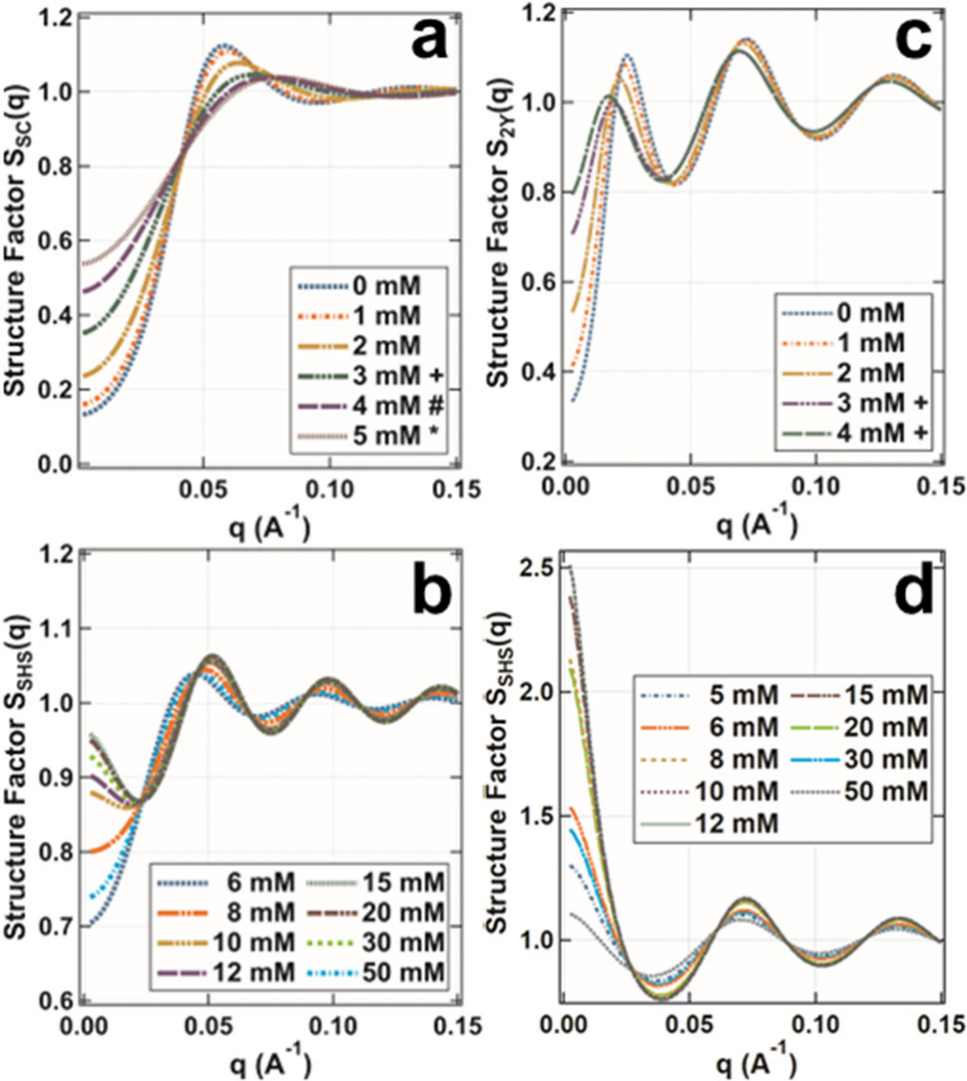
Structure factors calculated from Fig. 2 as a function of salt concentration. **a**, **b** show the structure factors for 80 mg/ml BSA while **c**, **d** show the structure factor for 80 mg/mL BLG. Note that the shown structure factors were calculated using different potentials. Thus, the SC potential was used for Figure **a**, while a 2Y potential (further information is given in the Supporting Information) was used for Figure **c**, and an SHS potential was used for Figures (b, c). For all the shown structure factors (**a**, **b**, **c**, **d**) the respective effective radius of a sphere was used which was previously calculated based on the axes of an oblate ellipsoid, used for SAXS data fitting (see Fig. 2) [78]. In **a**, all conditions were approximated using a particle size of 66.7 Å, exceptions were marked with special characters. Conditions marked with a + were approximated using a particle size of 67.97 Å, conditions marked with a # were approximated using a particle size of 68.75 Å, and conditions marked with a * were approximated using of 69.19 Å. In **c**, all conditions were approximated using a particle size of 50.41 Å, exceptions were marked with a + character. The conditions marked with a + were approximated using a particle size of 50.65 Å. In **b**, all conditions were approximated using a particle size of 69.83 Å, for **c** a particle size of 51.97 Å was used. For further information, consult the experimental section on SAXS data analysis as well as the Supporting Information

The effective structure factors *S*(*q*) for BSA and BLG, were calculated based on the fitting parameters (Fig. 2), which were previously converted from oblate ellipsoids to the respective effective radius of a sphere and subsequently depicted in Fig. 4.[78] In Fig. 4a the evolution of $S_{SC} \left( q \right)$ for BSA (80 mg/ml) for increasing salt concentrations is shown. The structure factor at $q = 0$ is equal to the normalized osmotic compressibility. A screened Coulombic structure factor of $S_{SC} \left( 0 \right) < 1$ indicates the dominance of the repulsive interaction. The first peak ($\sim 0.06$ Å^−1^) of $S_{SC} \left( q \right)$ represents the correlation between protein molecules in the solution. For increasing salt concentrations, the peak becomes broader and shifts its position toward higher *q* values, which suggests a decrease in the correlation length. Furthermore, $S_{SC} \left( {q \to 0} \right)$ increases with increasing salt concentrations. Therefore, an increase in salt concentration not only decreases the repulsive force, but also weakens the correlation between the protein molecules in the solution.

Figure 4c shows the evolution of $S_{2Y} \left( q \right)$ for BLG (80 mg/ml) for increasing salt concentrations. Starting from low *q*, the first of the two visible peaks ($\sim 0.03$ Å^−1^) represents the correlation of clusters formed whereas the second peak ($0.07$ Å^−1^) represents the correlation between protein molecules [48, 82]. With increasing salt concentration, the peak shifts toward lower *q* values ($\sim 0.02$ Å^−1^), which suggests the formation of small clusters with increasing size. This can be attributed to an increasing particle size, required to properly fit the data (Fig. 4c). The second peak ($\sim 0.07$ Å^−1^) represents the particle spacing. Due to the increased particle size induced by an increasing salt concentration, this peak shifts as well to lower *q* values indicating enlargement of particle spacing. Furthermore, $S_{2Y} \left( {q \to 0} \right)$ increases with increasing salt concentration, thus suggesting a decrease in the repulsive force.

In Fig. 4d, $S_{SHS} \left( {q \to 0} \right)$ increases up to a salt concentration of 12 mM, indicating a decreasing repulsive force. This finding is in line with the reduced second virial coefficient analysis as well as the $1{/}I\left( {q \to 0} \right)$ analysis (Fig. 3). A further increase in salt yields a decay in $S_{SHS} \left( {q \to 0} \right)$ suggesting an increasing repulsive force. Again, this observation is in line with the analysis of the reduced second virial coefficient analysis as well as the $1{/}I\left( {q \to 0} \right)$ analysis (Fig. 3). The same is true for BSA with salt concentrations above 5 mM (Fig. 4b); the only difference is that the strongest observed attraction is reached at 15 mM salt.

For both proteins, the first visible peak, $\sim 0.05$ Å^−1^ for BSA and $\sim 0.07$ Å^−1^ for BLG, increases in height and shifts its position toward higher *q* values, indicating higher correlation and a diminishing particle spacing, respectively (Fig. 4b and 4c). A similar analysis was carried out for the proteins OVA and HSA, which can be found in Figures S6 and S7 in the Supporting Information.

### Static and dynamic light scattering characterization of diffusion properties and interaction

**Fig. 5:**
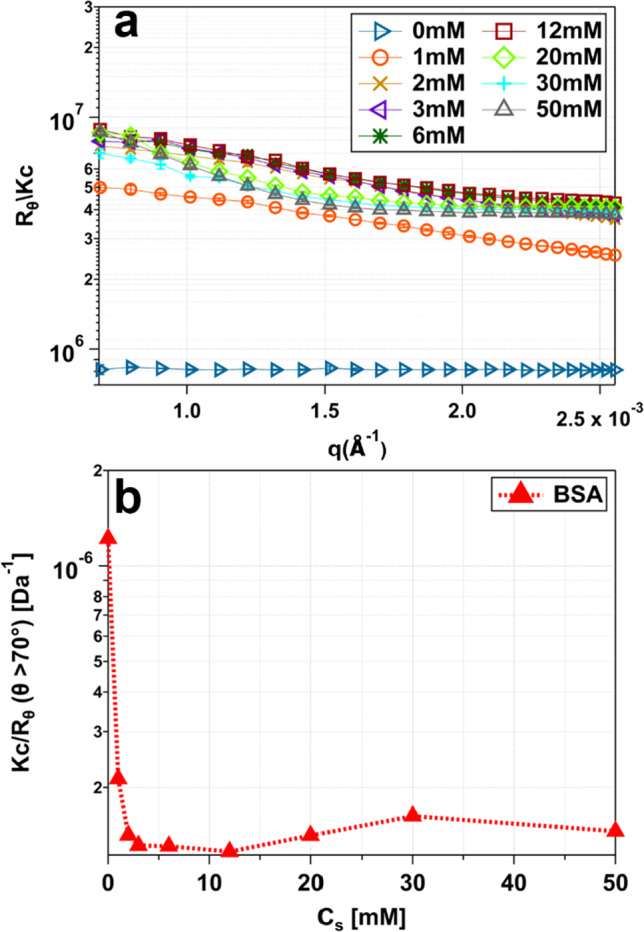
SLS intensity measurements **a** and inverse intensity Kc/${\text{R}}_{{\uptheta }}$, measured at a scattering angle between $70{ }^\circ$ and $90{ }^\circ$ **b**. For **a** and **b**, the measured samples contain 20 mg/ml BSA with increasing concentrations of Hac (0 mM to 50 mM). The shown lines (solid and dashed) represent guides to the eye. Note that error bars can be smaller than the symbols used for display

**Fig. 6:**
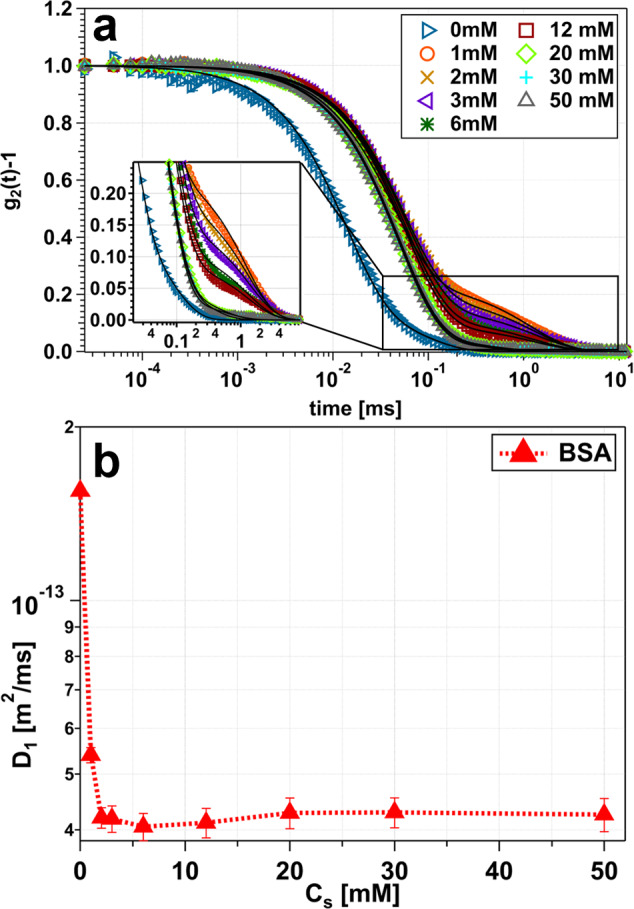
**a** Representative normalized autocorrelation functions (Eq. 5) as a function of decorrelation time for samples containing 20 mg/ml BSA with increasing concentrations of Hac (0–50 mM). The scattering angle was set to $\theta \ge 70^\circ$. The corresponding diffusion coefficient for the first of the two decays is depicted in **b**. The dashed lines represent guides to the eye. Note that error bars can be smaller than the symbols used for display

In the following, results obtained by SLS (Fig. 5) and DLS (Fig. 6) are discussed. Looking at the SLS analysis (Fig. 5) of 20 mg/ml BSA with increasing concentrations of Hac (0–50 mM), it becomes apparent that the low-*q* trend of the scattered intensities is comparable to the trend discussed for the SAXS analysis (see Fig. 3). With increasing salt concentration, the intensity increases as well. The highest intensity is visible at 12 mM. A further increase in the salt concentration yields in a low-*q* intensity decrease (Fig. 5a). This behavior of increasing and decreasing low-*q* intensities as a function of continuous increasing salt concentration is labeled reentrant interaction (RI). Moreover, small clusters with a broad range are visible for 1 mM, for higher salt concentrations (2–50 mM) the low-*q* intensity increase indicates the formation of bigger aggregates. Despite the presence of aggregates, no turbidity which would indicate a phase transition, was recognizable.

The SLS inverse intensity $Kc{/}R_{\theta }$ (Fig. 5b) features a comparable trend to the $1/I\left( {q \to 0} \right)$ SAXS data shown above (see Fig. 3b). The local minima can be seen at 12 mM salt which is an indicator for attractive conditions. For higher molarities, the intensity increases again, but with a much shallower slope. Again, this effect may be caused by screening effects of the ${\text{Cl}}^{ - }$ counterion. Nevertheless, differences between $1/I\left( {q \to 0} \right)$ behavior and the SLS inverse intensity $Kc{/}R_{\theta }$ are obvious. The most significant difference between the two is seen at low salt concentrations (0–3 mM), as the decreasing slope is much steeper for $KC{/}R_{\theta }$. However, the local minimum is within the same range for both measurements. It is important to note that the SAXS measurements were made with denser sampling (see Fig. 3b). Another deviation is observed at 30 to 50 mM. This difference could occur due to differences in sample preparation or fluctuations. Similar to above, we assume an uncertainty of 10% due to sample preparation and data acquisition.

In Fig. 6 the results for the DLS measurements are presented. The autocorrelation functions (Fig. 6a) of 20 mg/ml BSA with increasing salt concentrations (0–50 mM) were fitted (black lines) with a double exponential decay (see Eq. 5). Based on these fits the fast diffusion coefficient (D_1_) of the dominant dynamic mode was obtained (see Fig. 6b). However, two decays are visible (Fig. 6a), with the most pronounced double-exponential character to be seen for 1 mM Hac. Importantly, the second of the two mentioned decays correspond to a slower particle diffusion, meaning bigger particle sizes. Therefore, this decay can be assigned to aggregation, which is not within the scope of this paper.

A general trend of increasing decay times (0–1 mM) followed by decreasing decay times (1–50 mM) is to be seen (Fig. 6a). This trend of increasing and decreasing decay times can be ascribed to a RI behavior. Looking at the diffusion (Fig. 6b), the biggest decrease in the diffusion coefficient (*D*_1_) is to be seen within the range from 0 to 3 mM. Increasing the Hac concentration yields a local minimum at 6 mM Hac. Further increase in Hac concentration does not induce significant changes of the diffusion but results in a constant value (20–50 mM, Fig. 6b). The slower diffusion (decrease in the diffusion constant) indicates dimer and trimer formation, indicating an increasing particle size. Moreover, this is consistent with other data sets presented here (see Fig. 4).

Due to variations in the supplied BLG protein quality, a reliable DLS measurement was not possible and is therefore not shown.

### Discussion on the Hac-induced RC Behavior of DNA/RNA and the absence of RC for BLG, BSA, HSA, and OVA

The results presented above are compared to both: Studies investigating RC of negatively charged globular proteins in the presence of multivalent metal cations and studies examining RC of DNA or RNA in the presence of Hac.

First, looking at the phase behavior of negatively charged globular proteins in solution given the presence of multivalent cations, RC is observed [46]. The process of RC is characterized by a phase diagram featuring a sector consisting of aggregates (Regime II). With continuously increasing salt concentration at a fixed protein concentration, redissolution of the formed aggregates occurs [83]. The proteins BLG, BSA, HSA, and OVA showed RC in the presence of YCl_3_, LaCl_3_, FeCl_3_, and AlCl_3_, respectively [46]. The driving force of the RC is ascribed to both, the cation-mediated inversion of protein charge upon binding and the intermittent cation-mediated attraction [26]. Studies investigating RC, utilizing SAXS have shown that RC is associated with an increasing, followed by a decreasing, intensity at low *q* values, given a constant protein concentration combined with a continuously increasing salt concentration. The SHS fits enabling the determination of the reduced second virial coefficients showed a similar behavior [69, 70]. A high intensity at low *q* values (scattering profile) can be interpreted as attractive interaction, since the corresponding reduced second virial coefficient is negative and hence of attractive character. Therefore, the protein-salt system becomes more and more attractive with a maximum at a given salt concentration (highest low-*q* intensity in the scattering profiles with a corresponding minimum in the reduced second virial analysis). Typically, the highest attraction is found within the second regime as aggregates or clusters are dominating. The subsequent decrease in low *q* intensity indicates a weakening of the attraction accompanied by an increasing reduced second virial coefficients (transition from Regime II to Regime III) [69, 70]. The investigated protein-Hac systems, however, remained within Regime I (see Figs. 1 and 3). Nevertheless, the course of the scattering profiles and the reduced second virial coefficients are comparable, despite the missing optical phase transition to the second or third regime, respectively. Therefore, it can be assumed that the observed attraction is not sufficiently strong to induce the transition to the second regime.

Considering ion-induced protein interactions with regard to the different cation radii, it can be seen that the strength of attraction decreases with increasing cation radius [80]. Similar observations were made for HSA in the presence of different lanthanoid cations [84]. Hac features a 420 pm ion radius, whereas the ion radii of Ho^3+^ or Y^3+^ are 4.6 times smaller. The ion radius for La^3+^ is four times smaller compared to Hac [85]. A possible explanation for the decreasing ion-induced protein attractions with increasing ion radius could be that for small ion radii, the charge of the ion is concentrated near the protein binding site [80]. The results of the investigated proteins in the presence of Hac are in line with other studies [80, 84], since Hac, being the largest cation among those listed, evokes the least attraction. Hence, the ionic radius is one parameter influencing the ion-protein interaction.

**Fig. 7:**
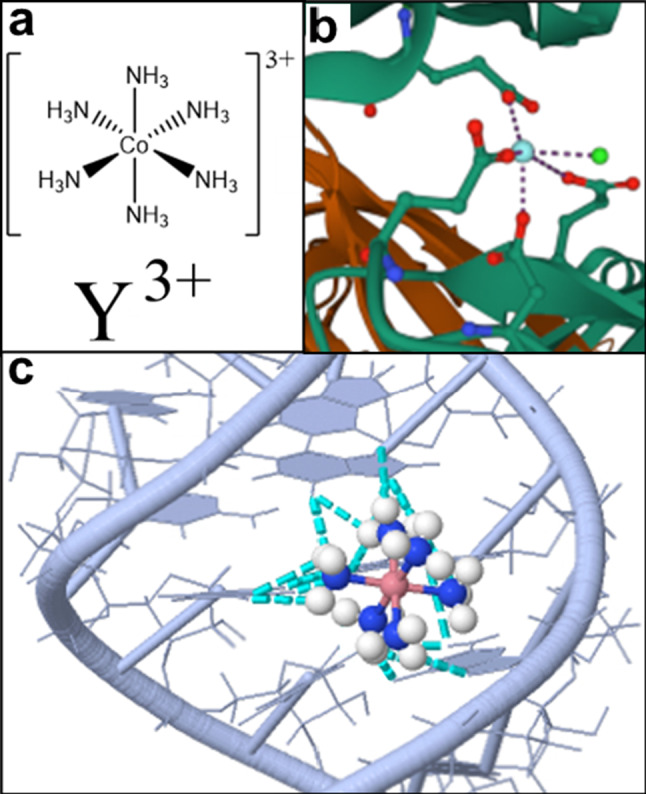
**a** Comparison of the two Lewis formulas of the hexamine cobalt(III) cation (top; **a**) and of yttrium cation (bottom; **a**). In both cases, the chloride counterions of were omitted to provide a simpler representation. **b** Ionic Y^3+^ bridging between two BLG monomers. The schematic was obtained by use of PDB entry 3PH5 [86], and the Mol* Viewer software [87]. **c** Schematic illustration of the P5b stem-loop group I intron ribozyme with the Hac binding sites highlighted. The central cobalt atom is shown in dark pink, while its ammonia ligands (NH_3_) are highlighted in blue (N) and white (H). The dashed cyan lines represent the established hydrogen bonds. The six ammonia ligands form hydrogen bonds with O6 and N7 of the three guanines (the right strand of the shown helix) and O4 of the two uracils (U13 and U14) located on the opposite (left) strand. The schematic was obtained using JSmol software [88] with PDB entry 1AJF [36]

However, structural differences between Hac and the listed cations have to be considered as well (see Fig. 7a). Hac features six covalently bonded ammonia ligands (NH_3_) arranged octahedrally around the central cobalt atom that do not exchange with the solvent, yielding a kinetically stable complex in aqueous solution (see Fig. 7a) [36, 37, 40]. This structural difference may also contribute to weaker ion-protein interactions, due to the fact that the charge of the ion may not be concentrated near the possible protein binding site.

Yet, Hac induces reentrant condensation of DNA and RNA, as demonstrated by several studies [30–34, 37]. However, Hac does not induce RC of negatively charged globular proteins as demonstrated by the experiments depicted here. The structural differences between DNA, RNA and proteins are of importance: proteins feature a complex surface pattern due to inhomogeneously distributed positive and negative charges. In addition, the binding sites feature different geometries resulting in few, but strong binding sites for interactions with multivalent cations [23]. In contrast, nucleic acids feature a more homogenous charge pattern. Typically, the prevalence of the phosphate backbone is responsible for the overall negative charge distribution [23]. Given these structural differences, the reason for RC in DNA and its absence in globular negatively charged proteins might be due to different cation binding or bridging mechanisms. Considering the mechanism of ion-activated attractive patches, solvent-exposed carboxyl side chains of negatively charged globular proteins located at the protein surface form coordinative bonds with multivalent metal ions such as Y^3+^.

This process is characterized by an activation and subsequent occupation of a protein patch. Every protein features only a limited number of patches on its surface. Only if an activated patch interacts with a non-activated (unoccupied) patch of a second protein, an ionic bridge, which connects the two protein molecules, can be established (see Fig. 7b) [26]. Therefore, a continuously increasing salt concentration increases the number of occupied patches, accompanied by an increasing number of ion salt bridges formed. Experimentally, the ionic bridge formation can be monitored by the charge inversion of the protein molecules [26]. For BLG in the presence of Y^3+^, the ion-activated attractive patch model is in good agreement as shown by crystal structure analysis. In this case, the four bound Y^3+^ ions contribute significantly to the bridging contacts of the unit cell (see Fig. 7b) [21, 86]. A different situation can be seen for HSA in the presence of Y^3+^. The majority of contacts are formed by protein–protein interactions, whereby the influence of the multivalent cation is noticeably weaker [25].

In comparison to the above, differences can be seen for the ion-induced DNA condensation. When modeling DNA condensation, given the assumption that the negative charge is solely compensated by Hac cations, the DNA “wraps” around the ion [32]. Additionally, the Hac molecule is kinetically stable, which implies that its six ammonia ligands are not replaced with the solvent. This feature allows the formation of hydrogen bonds between the six ammonia ligands and nucleic acids, with Hac being a so-called hydrogen donor (attributed to the present 18 hydrogen atoms). The major groove of DNA represents a typical yet specific binding site for Hac, as other possible binding sites are less frequently targeted. In the presence of a sufficiently high Hac concentration, the form of DNA is altered [35, 37] from B-DNA (right-handed helices, “average” DNA confirmation reported by Watson and Crick) to either A-DNA (slightly different confirmation but also right-handed helices) or Z-DNA (different DNA confirmation featuring left-handed helices) [37, 89]. Interestingly, the shape of Z-DNA offers a good fit of the Hac complex and allows the formation of five hydrogen bonds together with O6 guanine, N7 guanine and the present oxygen atoms of the phosphate backbone. In comparison, B-DNA does not offer these characteristics [37]. A-DNA features an easily accessible large major and a less pronounced minor groove compared to B-DNA. Within this major groove of A-DNA, two adjacent negatively charged phosphate backbones can be neutralized by the trivalent Hac cation, which may be the reason for the favoring of A-DNA [37]. Looking at an nuclear magnetic resonance study of the P5b stem-loop of the group I intron ribozyme RNA (see Fig. 7c), [36] the exact binding of the Hac-complex was elucidated. The major groove features not only a negatively charged surface but also several acceptors for hydrogen bond formation with the Hac ion [36]. The hydrogen bonds are formed between the Hac complex and O6 as well as N7 of the three guanines (G7, G6, and G5) and the O4 of the two uracil nucleobases (U13 and U14; see Fig. 7c) [36]. A similar behavior was found for NMR studies with group II intron Sc.aI5γ [35]. Here, the three determined Hac binding sites featured similar conditions of additional stabilization due to hydrogen bonds [35]. Therefore, these results suggests that the binding of Hac requires not only negative charges, but multiple hydrogen bonds to induce condensation. These requirements are fulfilled for DNA or RNA but are not met in the case of globular proteins.

## Conclusion

The comparison of several multivalent cations with Hac shows that Hac features by far the largest cation radius (420 pm). This is in line with an observed decreasing ion-induced protein interaction strength with increasing cation radius. Furthermore, the obtained reduced second virial coefficient analysis shows that none of the protein-Hac systems investigated induces strong enough attraction, resulting in the absence of LLPS due to insufficient attractions. This was also confirmed by SLS and DLS measurements, which indicate cluster formation and oligomerization (formation of dimers/trimers), but neither phase transition nor LLPS was observed.

Another possible aspect contributing to the RI behavior of globular negatively charged proteins (BLG, BSA, HSA, and OVA) in the presence of Hac could be the low charge density of Hac. This might occur due to widely distributed charges that act as single counterions, resulting in nonspecific binding of Hac which is sufficient to induce RI but insufficient to induce RC. Moreover, differences in the binding mechanism of multivalent metal ions to proteins and Hac to RNA/DNA become apparent. While the multivalent cations interact with the solvent-exposed carboxyl side chains of negatively charged globular proteins (e.g.,, BLG with Y^3+^), the Hac cation features additional hydrogen bonds when binding to a DNA groove. Therefore, the binding of the Hac cation to DNA is not only specific, but requires a precise fit, negative charges, and atoms to form hydrogen bonds with. This leads to the assumption that Hac mostly binds through hydrogen bonding. So far, however, all crystal structures of negatively charged proteins in the presence of multivalent cations resolved by our group indicate that the binding mechanism is of electrostatic nature, implying that net negatively charged residues complex the cation [21, 25, 90]. Hence, hydrogen bonding of Hac with the globular, net negatively charged proteins may occur. This is in turn supported by the herein presented experiments, such as the formation of clusters, confirmed by SLS and DLS and, the weak RI confirmed by SAXS. Therefore, this could be an explanation for the presence of RI and absence the of RC in the investigated protein-Hac systems.

### Supplementary Information

Below is the link to the electronic supplementary material.

Supplementary file1 (PDF 1107 KB)

## Data Availability

Raw and processed data are available free of charge upon reasonable request.

## Abbreviations

Hac: Hexamine cobalt(III) cation
RC: Reentrant condensation
LLPS: Liquid–liquid phase separation
BLG: Bovine β lactoglobulin
BSA: Bovine serum albumin
HSA: Human serum albumin
OVA: Albumin from hens chicken egg white
SAXS: Small-angle X-ray scattering
SLS: Static light scattering
DLS: Dynamic light scattering
MW: Molecular weight
SHS: Sticky hard sphere potential
2Y: Two Yukawa potential
SC: Screened Coulombic potential
HS: Hard sphere potential
$B_{2}/B_{2}^HS {B}_{2}/{B_{2}^{HS} }$: Reduced second virial coefficient
pI: Isoelectric point

## References

[1] Gunton JD, Shiryayev A, Pagan DL (2014). Protein Condensation.

[2] Vekilov PG (2004). Cryst. Growth Des..

[3] Piazza R (2000). Curr. Opin. Colloid Interface Sci..

[4] Ghiso J, Frangione B (2002). Adv. Drug Deliv. Rev..

[5] Da Fonseca M, Oueis HS, Casamassimo PS (2007). Pediatr. Dent..

[6] Ross CA, Poirier MA (2004). Nat. Med..

[7] Patel A, Lee HO, Jawerth L, Maharana S, Jahnel M, Hein MY, Stoynov S, Mahamid J, Saha S, Franzmann TM, Pozniakovski A, Poser I, Maghelli N, Royer LA, Weigert M, Myers EW, Grill S, Drechsel D, Hyman AA, Alberti S (2015). Cell.

[8] Wang Y, Lomakin A, McManus JJ, Ogun O, Benedek GB (2010). Proc. Natl. Acad. Sci. U.S.A..

[9] Siezen RJ, Fisch MR, Slingsby C, Benedek GB (1985). Proc. Natl. Acad. Sci. U.S.A..

[10] Muschol M, Rosenberger F (1997). J. Chem. Phys..

[11] Galkin O, Chen K, Nagel RL, Hirsch RE, Vekilov PG (2002). Proc. Natl. Acad. Sci. U.S.A..

[12] Soraruf D, Roosen-Runge F, Grimaldo M, Zanini F, Schweins R, Seydel T, Zhang F, Roth R, Oettel M, Schreiber F (2014). Soft Matter.

[13] Piazza R (2004). Curr. Opin. Colloid Interface Sci..

[14] Zhang F, Skoda MWA, Jacobs RMJ, Zorn S, Martin RA, Martin CM, Clark GF, Weggler S, Hildebrandt A, Kohlbacher O, Schreiber F (2008). Phys. Rev. Lett..

[15] Platten F, Hansen J, Wagner D, Egelhaaf SU (2016). J. Phys. Chem. Lett..

[16] Hansen J, Platten F, Wagner D, Egelhaaf SU (2016). Phys. Chem. Chem. Phys..

[17] Hagen MHJ, Frenkel D (1994). J. Chem. Phys..

[18] ten Wolde PR, Frenkel D (1997). Science.

[19] Noro MG, Frenkel D (2000). J. Chem. Phys..

[20] Matsarskaia O, Braun MK, Roosen-Runge F, Wolf M, Zhang F, Roth R, Schreiber F (2016). J. Phys. Chem. B.

[21] Zhang F, Zocher G, Sauter A, Stehle T, Schreiber F (2011). J. Appl. Crystallogr..

[22] Wolf M, Roosen-Runge F, Zhang F, Roth R, Skoda MW, Jacobs RM, Sztucki M, Schreiber F (2014). J. Mol. Liq..

[23] Matsarskaia O, Roosen-Runge F, Schreiber F (2020). Chemphyschem Eur. J. Chem. Phys. Phys. Chem..

[24] Fries MR, Stopper D, Braun MK, Hinderhofer A, Zhang F, Jacobs RMJ, Skoda MWA, Hansen-Goos H, Roth R, Schreiber F (2017). Phys. Rev. Lett..

[25] Maier R, Zocher G, Sauter A, Da Vela S, Matsarskaia O, Schweins R, Sztucki M, Zhang F, Stehle T, Schreiber F (2020). Cryst. Growth Des..

[26] Roosen-Runge F, Zhang F, Schreiber F, Roth R (2014). Sci. Rep..

[27] Roosen-Runge F, Heck BS, Zhang F, Kohlbacher O, Schreiber F (2013). J. Phys. Chem. B.

[28] Akiyama R, Sakata R (2011). J. Phys. Soc. Jpn..

[29] Fujihara S, Akiyama R (2014). J. Mol. Liq..

[30] Widom J, Baldwin RL (1980). J. Mol. Biol..

[31] Nguyen TT, Rouzina I, Shklovskii BI (2000). J. Chem. Phys..

[32] Todd BA, Rau DC (2008). Nucleic Acids Res..

[33] Pelta J, Livolant F, Sikorav JL (1996). J. Biol. Chem..

[34] Deng H, Bloomfield VA (1999). Biophys. J..

[35] Donghi D, Pechlaner M, Finazzo C, Knobloch B, Sigel RKO (2013). Nucleic Acids Res..

[36] Kieft JS, Tinoco I (1997). Structure.

[37] Rowinska-Zyrek M, Skilandat M, Sigel RKO (2013). Z. Anorg. Allg. Chem..

[38] Bougie I, Bisaillon M (2009). Biochem. Biophys. Acta..

[39] Hanahan D (1983). J. Mol. Biol..

[40] Chang EL, Simmers C, Knight DA (2010). Pharmaceuticals.

[41] Cunat P-J (2004). Euro Inox.

[42] Hedberg YS, Odnevall-Wallinder I (2016). Biointerphases.

[43] Milošev I (2017). Corrosion.

[44] Hedberg YS, Pettersson M, Pradhan S, Odnevall-Wallinder I, Rutland MW, Persson C (2015). ACS Biomater. Sci. Eng..

[45] Chen JK, Thyssen JP (2018). Metal Allergy: from Dermatitis to Implant and Device Failure.

[46] Zhang F, Weggler S, Ziller MJ, Ianeselli L, Heck BS, Hildebrandt A, Kohlbacher O, Skoda MWA, Jacobs RMJ, Schreiber F (2010). Proteins.

[47] Verheul M, Pedersen JS, Roefs SPFM, de Kruif KG (1999). Biopolymers.

[48] Braun MK, Grimaldo M, Roosen-Runge F, Hoffmann I, Czakkel O, Sztucki M, Zhang F, Schreiber F, Seydel T (2017). J. Phys. Chem. Lett..

[49] Naqvi Z, Ahmad E, Khan RH, Saleemuddin M (2013). Cell Biochem. Biophys..

[50] Yang M, Dutta C, Tiwari A (2015). J. Phys. Chem. B.

[51] Peters T (1996). All about Albumin: Biochemistry, Genetics, and Medical Applications.

[52] Nisbet AD, Saundry RH, Moir AJ, Fothergill LA, Fothergill JE (1981). Eur. J. Biochem..

[53] Pervaiz S, Brew K (1985). Science.

[54] Hirayama K, Akashi S, Furuya M, Fukuhara K-I (1990). Biochem. Biophys. Res. Commun..

[55] Martos G, Contreras P, Molina E, López-Fandiño R (2010). J. Agric. Food Chem..

[56] Beeley JA, Stevenson SM, Beeley JG (1972). Biochim. Biophys. Acta Protein Struct..

[57] Elofsson UM, Paulsson MA, Arnebrant T (1997). Langmuir.

[58] Ianeselli L, Zhang F, Skoda MWA, Jacobs RMJ, Martin RA, Callow S, Prévost S, Schreiber F (2010). J. Phys. Chem. B.

[59] Lee JC, Timasheff SN (1974). Biochemistry.

[60] Hianik T, Ponikova S, Bagelova J, Antalik M (2006). Bioorg. Med. Chem. Lett..

[61] Babul J, Stellwagen E (1969). Anal. Biochem..

[62] Lundblad RL, Macdonald F (2018). Handbook of Biochemistry and Molecular Biology.

[63] Zhang XH, Zhang XD, Lou ST, Zhang ZX, Sun JL, Hu J (2004). Langmuir.

[64] Mirtallo JM, Caryer K, Schneider PJ, Ayers L, Fabri PJ (1981). Am. J. Health Syst. Pharm..

[65] Sigma Aldrich, Merck KGaA: Darmstadt, Germany, Hexaamminecobalt(III) chloride, 99%; CAS-RN: 10534-89-1; Product no: 481521 (2021), https://www.sigmaaldrich.com/DE/de/product/aldrich/481521. Accessed 30 Mar 2021

[66] Blanchet CE, Spilotros A, Schwemmer F, Graewert MA, Kikhney A, Jeffries CM, Franke D, Mark D, Zengerle R, Cipriani F, Fiedler S, Roessle M, Svergun DI (2015). J. Appl. Crystallogr..

[67] Baxter RJ (1968). J. Chem. Phys..

[68] Kline SR (2006). J. Appl. Crystallogr..

[69] Braun MK, Sauter A, Matsarskaia O, Wolf M, Roosen-Runge F, Sztucki M, Roth R, Zhang F, Schreiber F (2018). J. Phys. Chem. B.

[70] Braun MK, Wolf M, Matsarskaia O, Da Vela S, Roosen-Runge F, Sztucki M, Roth R, Zhang F, Schreiber F (2017). J. Phys. Chem. B.

[71] Menon SVG, Manohar C, Rao KS (1991). J. Chem. Phys..

[72] Vliegenthart GA, Lekkerkerker HNW (2000). J. Chem. Phys..

[73] Li Y, Lubchenko V, Vekilov PG (2011). Rev. Sci. Instrum..

[74] Schärtl W (2007). Light scattering from polymer solutions and nanoparticle dispersions.

[75] Zemb T, Lindner P (2002). Neutron X-rays and Light. Scattering Methods Applied to Soft Condensed Matter.

[76] Berne BJ, Pecora R (2013). Dynamic Light Scattering.

[77] Sinha SK, Jiang Z, Lurio LB (2014). Adv. Mater..

[78] Zhang F, Skoda MWA, Jacobs RMJ, Martin RA, Martin CM, Schreiber F (2007). J. Phys. Chem. B.

[79] Zhang F, Roosen-Runge F, Sauter A, Roth R, Skoda MWA, Jacobs RMJ, Sztucki M, Schreiber F (2012). Faraday Discuss..

[80] Matsarskaia O, Roosen-Runge F, Lotze G, Möller J, Mariani A, Zhang F, Schreiber F (2018). Phys. Chem. Chem. Phys..

[81] Hansen J-P (2006). Theory of Simple Liquids.

[82] Liu Y, Porcar L, Chen J, Chen W-R, Falus P, Faraone A, Fratini E, Hong K, Baglioni P (2011). J. Phys. Chem. B.

[83] Lobaskin V, Qamhieh K (2003). J. Phys. Chem. B.

[84] Schomäcker K, Mocker D, Münze R, Beyer G-J (1988). Int. J. Radiat. Appl. Instrum. Part A Appl. Radiat. Isot..

[85] Marcus Y (1997). Ion Properties.

[86] Zocher G, Stehle T (2011). J. Appl. Crystallogr..

[87] Sehnal D, Bittrich S, Deshpande M, Svobodová R, Berka K, Bazgier V, Velankar S, Burley SK, Koča J, Rose AS (2021). Nucleic Acids Res..

[88] Hanson RM, Prilusky J, Renjian Z, Nakane T, Sussman JL (2013). Isr. J. Chem..

[89] Ussery DW (2002). Encycl. Life Sci..

[90] Sauter A, Oelker M, Zocher G, Zhang F, Stehle T, Schreiber F (2014). Cryst. Growth Des..

