# Supplementary material for: Effective interactions and phase behavior of protein solutions in the presence of hexamine cobalt(III) chloride
Source: Eur Phys J E Soft Matter. 2023 Dec 5;46(12):119. doi: 10.1140/epje/s10189-023-00376-6 (PMC10698144; doi:10.1140/epje/s10189-023-00376-6)
Supplement: Supplementary file 1 — Supplementary file1 (PDF 1107 KB) [file 10189_2023_376_MOESM1_ESM.pdf]

# Supporting Information: Effective interactions and phase behavior of protein solutions in the presence of hexamine cobalt (III) chloride

Maximilian D. Senft [1*], Ralph Maier [1], Anusha Hiremath [1], Fajun Zhang [1*] and Frank Schreiber [1]

[1]Institut für Angewandte Physik, Universität Tübingen, Auf der Morgenstelle 10, 72076 Tübingen, Germany

*** E-mail: fajun.zhang@uni-tuebingen.de; maximilian.senft@uni-tuebingen.de**

## Small angle scattering data analysis

Small angle X-ray scattering (SAXS) was used in this work to investigate the protein-protein interactions induced by different ionic strengths of the trivalent metal salt hexamine cobalt (III) chloride (Hac) in aqueous solution. In the following, the SAXS data analysis is briefly described followed by a description of the used interaction potentials. For a non-spherical polydisperse system, given the scattering angle of $2\theta$, the static scattering intensity $I(q)$ as a function of the scattering vector $q = \frac{4\pi}{\lambda}\sin(\theta)$ can be defined as

$$I(q) = N_p(\Delta\rho)^2 V_p^2 P(q) S(q) \quad (1)$$

Here, $N_P$ denotes the total number of protein molecules per unit volume within the investigated solution. $V_P$ indicates the volume for a single protein. The scattering contrast $\Delta\rho = (\rho_P - \rho_S)$ represents the difference in electron density between protein molecules $(\rho_P)$ and the solvent $(\rho_S)$.[1–6] The form factor $P(q)$(Eq. 2.1) describes the shape of a protein molecule after orientational averaging. In this work an ellipsoidal form factor was used to describe the global shape of proteins and to fit the obtained data (eq. 2.1 and 2.2).[1, 2, 4, 6, 7]

$$P(q) \equiv \langle |F(q)|^2 \rangle = \int_0^1 dx \left| \frac{3(\sin u - u\cos u)}{u^3} \right|^2 \quad (2.1)$$

$$u = qb[(a/b)^2 x^2 + (1-x)^2]^{1/2} \quad (2.2)$$

The structure factor $S(q)$ contains information about the spatial arrangement, and the protein interactions.[2, 8] In the presented work, the protein solutions studied are monodisperse, but feature an elliptical rather than a spherical shape. In order to calculate the effective structure factor $\overline{S}(q)$, the volume of the ellipsoid is equated to that of a sphere with a similar 2nd virial coefficient.[9] In the following $S(q)$ is used to represent $\overline{S}(q)$. In addition, the Fourier transformation of the spherically averaged pair correlation function $g(r)$ is related to $S(q)$.[1, 2, 6, 8]

$$S(q) = 1 + N_P \int 4\pi r^2 [g(r) - 1] \frac{\sin(qr)}{qr} dr \quad (3)$$

Solving the Ornstein-Zernicke (OZ) equation (Eq. 4) using the rescaled mean spherical approximation closure relation, yields the total correlation function $h(r) = g(r) - 1$.

Importantly, the OZ equation decomposes the correlation into two parts: an indirect and a direct part.[2, 8]

$$h(|\vec{r_i} - \vec{r_k}|) = c(|\vec{r_i} - \vec{r_k}|) + \rho \int d^3 r_j c(|\vec{r_i} - \vec{r_k}|) h(|\vec{r_i} - \vec{r_k}|) \quad (4)$$

The distance between a pair of particles is referred to as $r_{i,k}$, and the total correlation function is represented by $h(r)$. However, to solve the OZ equation it is necessary to relate the direct

correlation function $c(r)$ with the interaction potential $u(r)$. This process is commonly termed closure relation. In the following, the interaction potentials used are described.[2, 8]

The sticky hard spheres interaction potential (SHS) is already described in detail within the main manuscript (view the experimental section).

**Screened coulombic potential (SC)**

This potential was used for BSA, OVA and HSA solutions with an Ionic strength less than $I < 5\,mM$. The potential can be defined as follows:

$$U_{SC}(r) = \begin{cases} \infty & 0 < r < \sigma \\ \pi\varepsilon_0\varepsilon\sigma^2\psi_0^2 \exp[-\kappa(r-\sigma)]/r & r > \sigma \end{cases} \tag{5}$$

here $\varepsilon_0$ corresponds to the permittivity of the free space, $\varepsilon$ represents the dielectric constant of the solvent, $\sigma$ represents the particle diameter, $\psi_0$ corresponds to the surface potential and the inverse Debye-Hückel screening length is represented by $\kappa$. Here $r$ represents the inter particle spacing.[7, 10, 11] By means of the (rescaled) mean spherical approximation (MSA), the structure factor for the SC potential was derived.

**Two Yukawa potential (2Y)**

The two Yukawa potential (2Y) extends the SC potential, as it features an attractive potential term ($K_2$) besides a repulsive exponential term ($K_1$). Again, utilizing the MSA closure relation it is possible to derive S(q) for a monodisperse elliptical particle system.[12] In this work, the 2Y potential was used for samples containing BLG admixed with Hac at low ionic strengths with $I < 5\,mM$.

$$\beta U_{2Y}(r) = \begin{cases} \infty & 0 < r < \sigma \\ -K_1 \frac{exp[-Z_1(r-\sigma)]}{(r/\sigma)} - K_2 \frac{exp[-Z_2(r-\sigma)]}{(r/\sigma)} & r > \sigma \end{cases} \tag{6}$$

With $\beta = 1/k_BT$.

**Hard sphere potential (HS)**

Given the condition of low to moderate ionic strengths, the surface charge of the proteins can be considered as screened. Therefore, the total interaction between the proteins is weak, as the main interaction is facilitated through excluded volume effects also known as hard sphere interactions.[4, 7]

$$U_{HS}(r) = \begin{cases} \infty, & r < \sigma \\ 0, & r \geq \sigma \end{cases} \tag{7}$$

Here, $\sigma$ denotes the particle diameter whereas *r* denotes the radius. At moderate ionic strength, the surface charge is sufficiently screened. The overall interparticle interaction is rather weak, and the protein molecules interact with each other mainly through hard-sphere (excluded volume effect) interactions.

In this case, the OZ equation is solved by using the Percus Yevick (PY) closure relation,[4, 7]

## Additional information on the used parameters for SAXS data analysis and fitting

The following two Tables S1 and S2 summarize the used particle size (Table S2) and scattering length density (Table S2). In Table S2 the proteins with the corresponding entry number and the calculated SLDs are listed.

Table S1: Particle sizes used (in Å) subdivided according to protein and the used potential for approximation. The particles were assumed to be elliptical.

| Potential | Protein | Axis a [Å] | Axis b [Å] |
|---|---|---|---|
| SC | OVA | 44.20 | 20.20 |
| HS | OVA | 53.65 | 19.82 |
| SHS | OVA | 57.00 | 19.82 |
| SC | HSA | 18.00 | 42.50 |
| SHS | HSA | 18.00 | 42.50 |

Table S2: Calculated scattering length density (Å$^{-2}$) listed together with the corresponding proteins.

| Protein | Calculated scattering length density [$\text{Å}^{-2}$] |
|---|---|
| BLG | $7.33 * 10^{-7}$ |
| BSA | $7.32 * 10^{-7}$ |
| OVA | $7.32 * 10^{-7}$ |
| HSA | $7.32 * 10^{-7}$ |

The protein volume fraction $\varphi$ can be calculated for any given protein concentration $c_p = m_p/V_s$ according to [13]

$$\varphi = \frac{c_p}{1+c_p \times \nu} \cdot \nu \tag{8}$$

Here, $c_p$ represents the protein concentration (g/ml), the protein mass is given by $m_p$, and $V_s$ denotes the volume of added water. $\nu$ corresponds to the specific volume (ml/g) of the respective protein. Based on equation (9), the respective protein volume fractions $\varphi$ were calculated for fitting the SAXS data obtained.

## Fitted SAXS data of Ovalbumin

The effective protein-protein interactions of the protein-salt solutions were investigated by use of systematic SAXS measurements. Figure S1 shows the representative SAXS measurements for 80 mg/ml OVA with increasing Hac concentrations (mM) in $H_2O$. Given low salt concentrations (0 to 4 mM), the net negative charges of the protein molecules dominate the effective protein-protein interactions, as illustrated by the correlation peak $q = 0.06\,Å^{-1}$. An increase in the salt concentration results in an increasing low q intensity, which indicates reducing repulsion, while concurrently, the correlation peak fades out (4 mM). As the salt concentration is increased further, the highest attraction becomes apparent at 30 mM salt. Any further increase of the salt concentration reduces the attractive strength. This is indicated by the decreasing low q intensity at 50 mM salt (Figure S1 b). For Ovalbumin at low salt concentrations (0 to 4 mM) the SC, for intermediate salt concentrations (5 to 8 mM) the HS and for high salt concentrations (10 to 50 mM) the SHS interaction potential was used (see solid black lines in Figure S1). According to table S2, the elliptical axes had to be adjusted for the approximation of the data using different potentials. One reason for this behavior could be dimerization of the ovalbumin monomers.

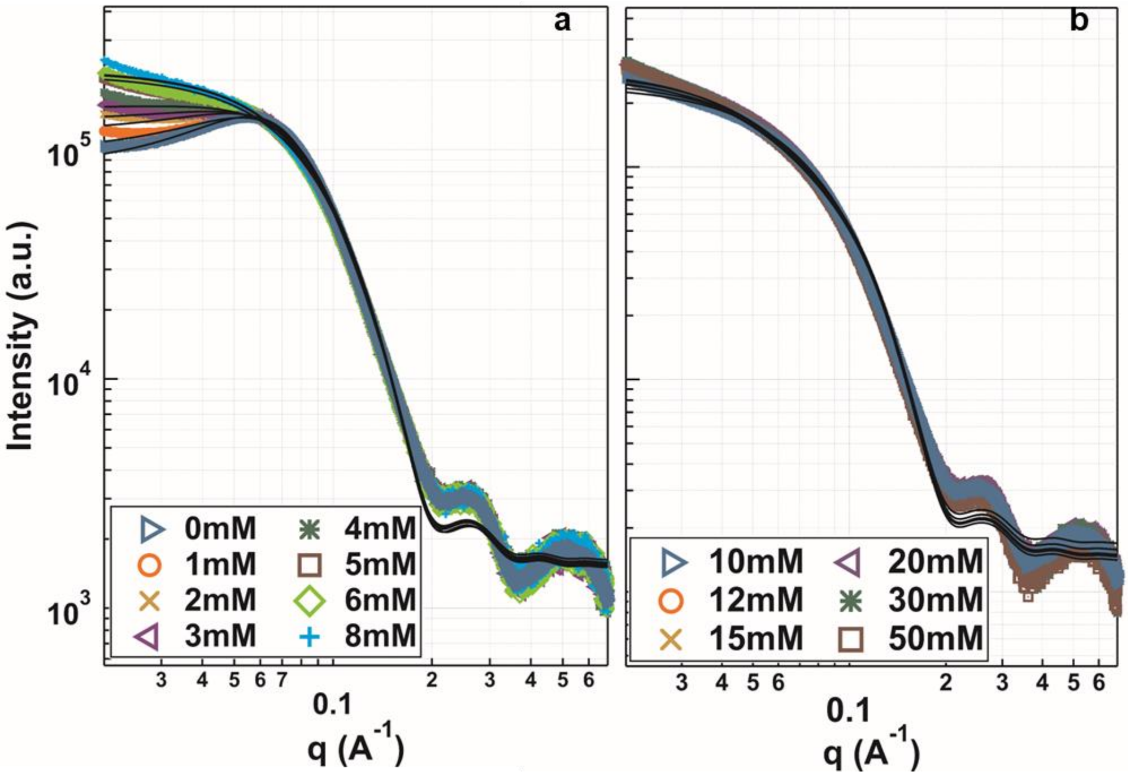


Figure S1: (a, b) SAXS data with model fits (solid black line) for samples in $H_2O$ containing 80 mg/ml OVA with increasing salt concentrations (0 to 50 mM). The scattering intensity at low $q$ increases with increasing salt concentration (a) and decreases and decreases in (b). In (a), for low ionic strengths (0 to 4 mM) the SC potential was used. For intermediate ionic strengths, (5 to 8 mM) the HS potential was consulted. The other remaining conditions (10 to 50 mM) were fitted by use of the ESHS potential (b). The respective error values are smaller than the markers used for illustration and are therefore not plotted for clarity.

## Fitted SAXS data of Human serum albumin

A similar evaluation of the SAXS data on the protein HSA with increasing salt concentrations of Hac was carried out. Figure S2 shows the representative SAXS measurements for 80 mg/ml OVA with increasing Hac concentrations (mM) in $H_2O$. Given low salt concentrations (0 to 5 mM), the net negative charges of the protein molecules dominate the effective protein-protein interactions, as illustrated by a correlation peak, visible at $q = 0.06\ \text{Å}^{-1}$. Increasing the salt concentration further, results in a vanished correlation peak. The highest low q intensity is visible at 8 mM. Increasing the salt concentration further results in a discontinuous decrease in low q intensity. Discontinuous, because the low q intensity for 12 mM salt is below the low q intensities for 10-, 15- and 20-mM salt. For HSA, at low salt concentrations (0 to 5 mM) the SC, for higher salt concentrations (6 to 30 mM) the SHS interaction potential was used (see solid black lines in Figure S2). The elliptical axes were fixed to the values given in Table S1.

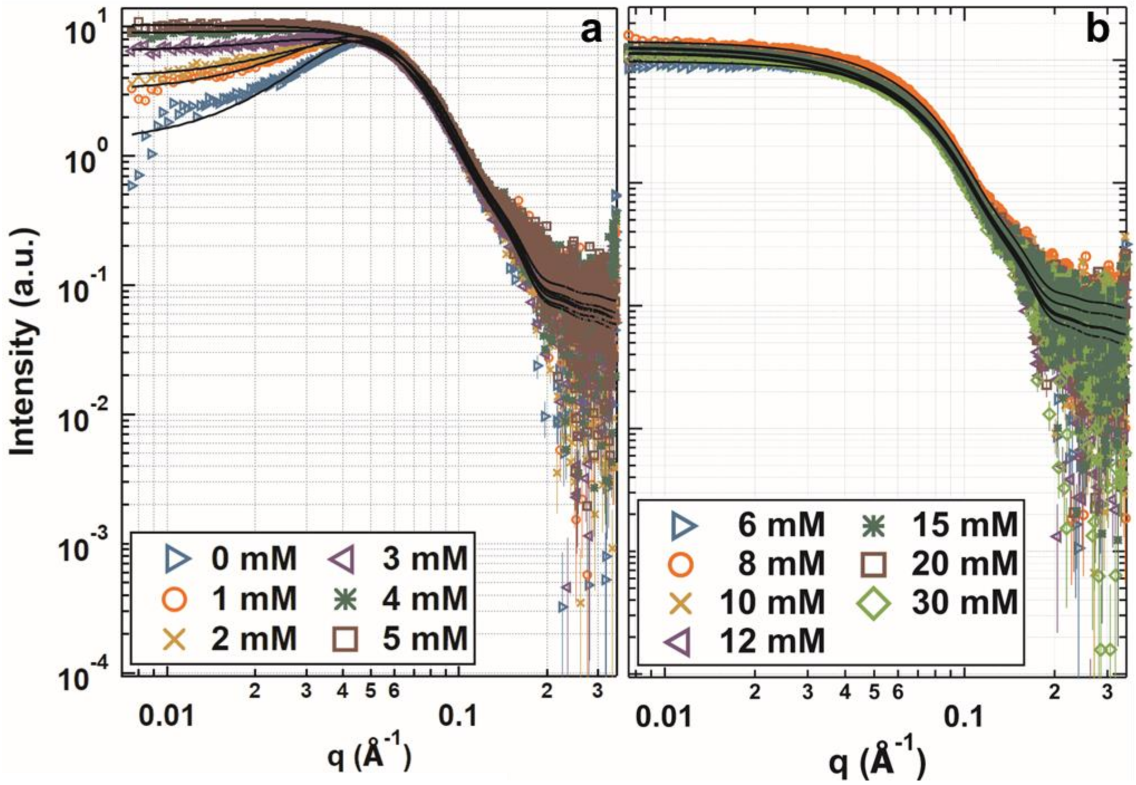


Figure S2: (a, b) Laboratory SAXS data with model fits (solid black line) for samples in $H_2O$ containing 80 mg/ml HSA with increasing salt concentrations (0 to 30 mM). The scattering intensity at low *q* increases with increasing salt concentration (a) and decreases and decreases in (b). In (a), for low ionic strengths (0 to 5 mM) the SC potential was used. The other remaining conditions (10 to 30 mM) were fitted by use of the ESHS potential (b). Note that the data from the laboratory SAXS device (device settings can be found in the Experimental section of the Manuscript) features a different *q*-range. Error bars are indicated by vertical lines.

## Second virial coefficient analysis of Ovalbumin and Human serum albumin

In Figure S3, the calculated second virial coefficient ($B_2/B_2^{HS}$) for the SAXS measurements of 80 mg/ml OVA and HSA in the presence of Hac are shown. For OVA (red triangles, Figure S3) it can be seen that form 10 mM on the second virial coefficient decreases rapidly. The minimum is reached at 30 mM salt. Taken together, the OVA Hac system remains repellent as the reduced second virial coefficient is greater than zero ($B_2/B_2^{HS} > 0$).

In the case of HSA (blue diamonds, Figure S3), starting from a low salt concentration (6 mM), the second virial coefficient decreases with increasing salt concentration. Increasing the salt concentration up to 10 mM results in weakening of the repulsions. At 10 mM a secondary minimum can be seen. Increasing the salt concentration up to 12 mM results in an increase of the reduced second virial coefficient. The primary minimum is visible at 15 mM salt. Further increase in salt concentration leads to an increased repulsion. Overall, the investigated HSA Hac system remains repellent as indicated by the second virial coefficient ($B_2/B_2^{HS} > 0$).

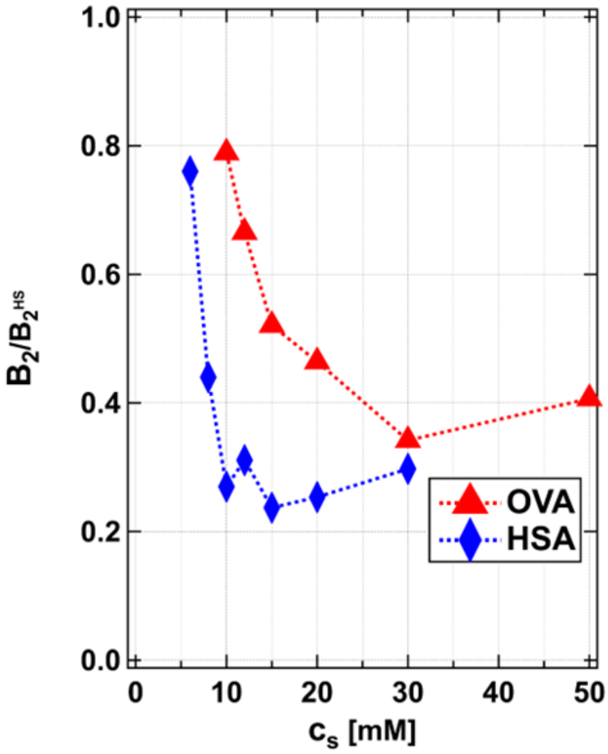


Figure S3: Reduced second virial coefficient behavior of the investigated OVA and HSA samples with a $c_p$ of 80 mg/ml and varying $c_s$ (mM), based on the ESHS potential fits presented in Figure S1 and S3. The respective error values are smaller than the markers used for illustration and are therefore not plotted for clarity. The dashed lines are guides to the eye.

## The inverse intensity

Importantly, this approach is connected to the reduced second virial coefficient via the isothermal compressibility $\chi_T$. The expansion of the osmotic pressure $\Pi$ for proteins in solution, by means of the protein number density $\rho$, allows to relate the virial coefficient.

$$\frac{\Pi}{\rho k_B T} = 1 + B_2\rho + \ \dots \tag{9}$$

The second virial coefficient $B_2$ is defined as

$$B_2 = -\frac{1}{2}\int \left(1 - e^{-\left(\frac{u(r)}{k_B T}\right)}\right) dr \tag{10}$$

Considering the structure factor in terms of the isothermal compressibility $\chi_T$ yields

$$I(q \to 0) \propto S(q \to 0) = \rho k_B T \chi_T \tag{11}$$

Further details are to be found in Braun et.al 2018. [14]

## Inverse intensity analysis of Ovalbumin

In Figure S4 the $1/I(q \to 0)$ trend for 80 mg/ml OVA admixed with increasing concentrations of Hac is shown. The inverse intensity follows the same trend as indicated by the reduced second virial coefficient analysis (see Figure S3), with the highest attractiveness at 30 mM salt.

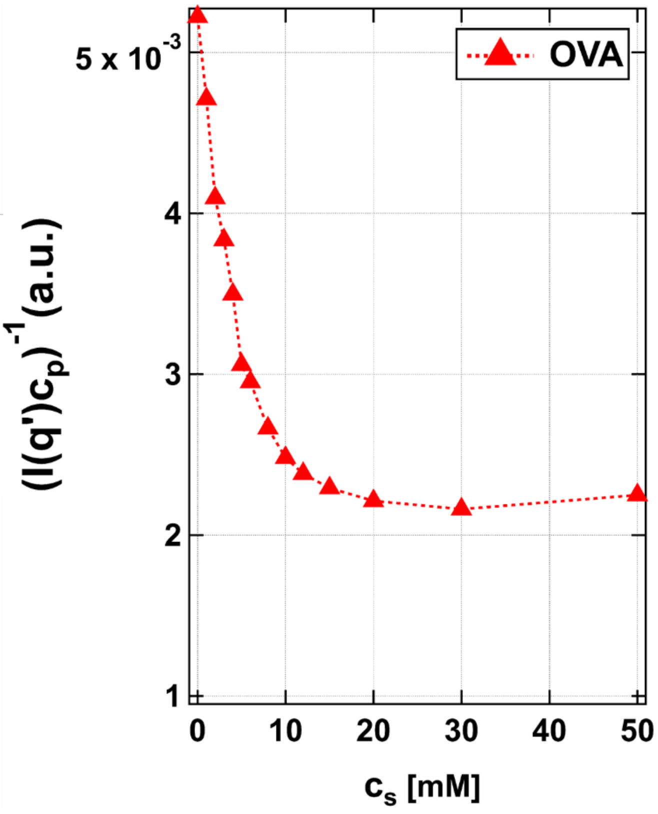


Figure S4: $1/I(q \to 0)$ behavior of the investigated OVA samples with $c_p$ 80 mg/ml and varying $c_s$ (0 to 50 mM Hac). The inverse intensities at $q' = 0.03\text{Å}^{-1}$ are normalized to the molecular weight of the protein monomer. The error values are smaller than the markers used for illustration and are therefore not plotted for clarity. The dashed lines are guides to the eye.

## Inverse intensity analysis of Human serum albumin

Looking at the fits from Figure S2, it can be seen that these describe the data only partially well. Deviations are visible at the intermediate (0.04 to 0.06 $Å^{-1}$) and high q (0.2 to 0.3 $Å^{-1}$) regions. From this it can be deduced that there are possible discrepancies between the virial coefficient analysis, which implies a model-based fitting of the data, and the model free $1/I(q \rightarrow 0)$ analysis. Note that the intensities for OVA (Figure S4) are different, due to the different devices used. Therefore, the intensity is given in arbitrary units (a.u.).

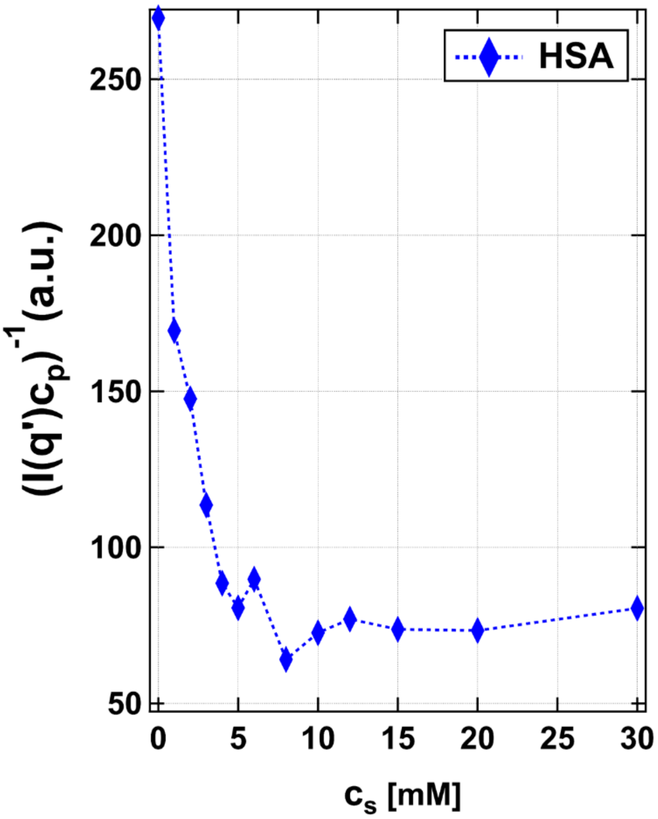


Figure S5: $1/I(q \rightarrow 0)$ behavior of the investigated HSA samples with $c_p$ 80 mg/ml and varying $c_s$ (0 to 30 mM Hac). The inverse intensities at $q' = 0.03Å^{-1}$ are normalized to the molecular weight of the protein monomer. The respective error values are smaller than the markers used for illustration and are therefore not plotted for clarity. The dashed lines are guides to the eye. Note that the data from the laboratory SAXS device (device settings can be found in the Experimental section of the Manuscript) features a different $q$-range.

## S(q) Data on Ovalbumin HSA

Figure S6 shows the *S(q)* for 80 mg/ml OVA with increasing Hac concentrations based on the fits provided in Figure S1. The effective structure factors *S(q)* were calculated based on the fitting parameters (see Table S1 and S2), which were previously converted from oblate ellipsoids to the respective effective radius of a sphere and subsequently depicted in Figure S6. A structure factor of $S_{SC}\ (0)\ <\ 1$ (see figure S6 a) indicates the dominance of repulsive interaction. The first peak $(\sim 0.075\ \text{Å}^{-1})$ of $S_{SC}$(q) represents the correlation between a pair of protein molecules in the solution. For increasing salt concentrations, the peak becomes broader and shifts its position towards higher q values, suggesting a decreasing correlation length. As Table S1 shows, the particle diameter was increased at intermediate and high ionic strengths (see Figure S6 b and c). The peak position shifted to lower q values $(\sim 0.05\ \text{Å}^{-1}$ ; see Figure S6 b). Looking at the $S(q)_{SHS}$, the first visible peak, $(\sim\ 0.05\ \text{Å}^{-1})$, increases in height and shifts its position towards higher $q$ values, which is an indication for a higher correlation and a diminishing particle spacing (Figure S6 c).

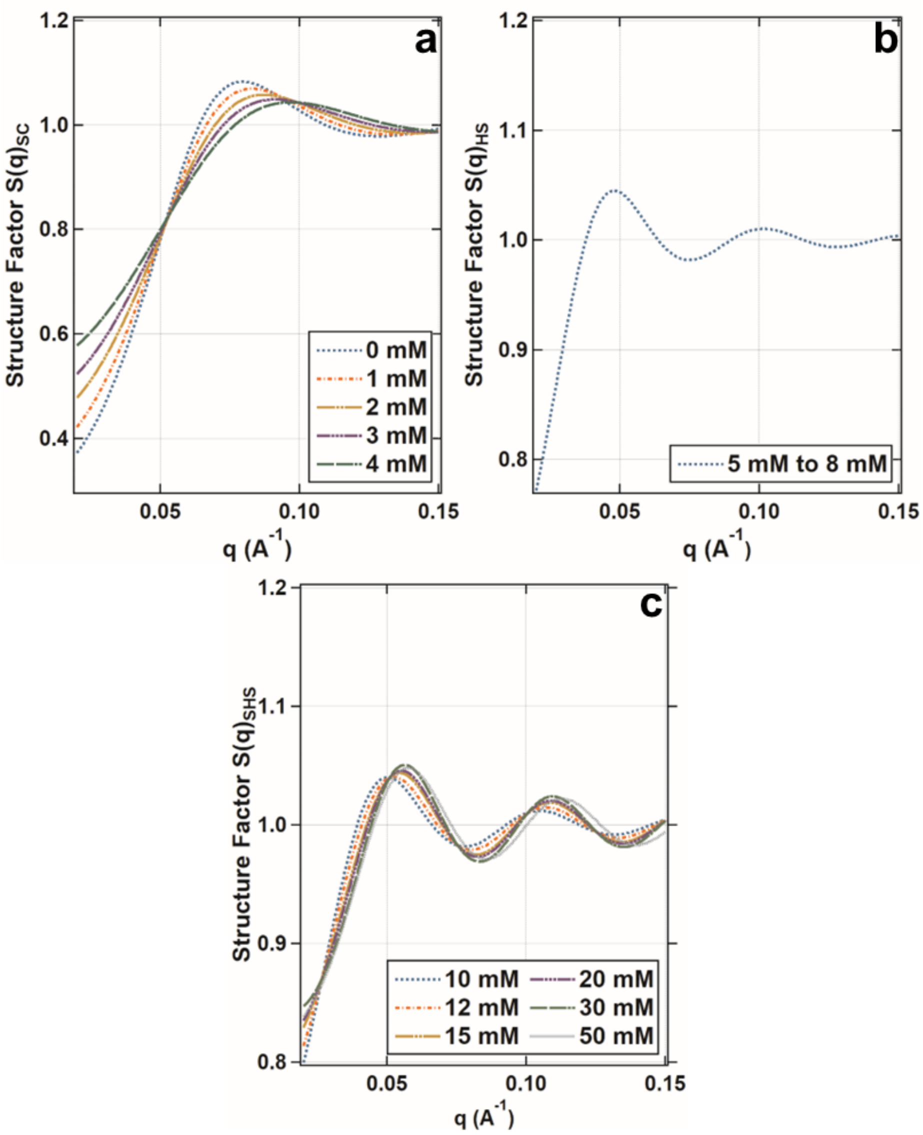


Figure S6: Structure factors calculated from Figure S1, as a function of the salt concentration. (a, b, and c) present the structure factors for 80 mg/ml OVA. Note that the shown structure factors were calculated using different potentials. Thus, the SC potential was used for figure (a), while a HS potential was used for figure (b) and an ESHS potential was used for figures (c). In (a), all conditions were approximated using a particle size of 55.33 Å, in (b) all conditions were approximated using a particle size of 60.18 Å. In (c), all conditions were approximated using a particle size of 62.04 Å. Note, that for all the shown structure factors (a, b, c, d) the respective effective radius of a sphere was used which was previously calculated based on the oblate ellipsoidal axes, used for SAXS data fitting.[4]

## S(q) Data on HSA

Figure S6 shows the *S(q)* for 80 mg/ml HSA with increasing Hac concentrations based on the fits provided in Figure S2. A structure factor of $S_{SC}\ (0)\ <\ 1$ (see figure S7 a) indicates the dominance of repulsive interaction. The first peak $(\sim 0.06\ \text{Å}^{-1})$ of $S_{SC}$(q) represents the correlation between a pair of protein molecules in the solution. For increasing salt concentrations, the peak becomes broader and shifts its position towards higher q values, suggesting a decreasing correlation length. As Table S1 shows, the particle diameter was increased towards higher high ionic strengths. Looking at the $S(q)_{SHS}$, the peak position shifted to lower q values $(\sim 0.05\ \text{Å}^{-1})$, and increases in height and shifts its position towards higher *q* values, which is an indication for a higher correlation and a diminishing particle spacing (Figure S7 b).

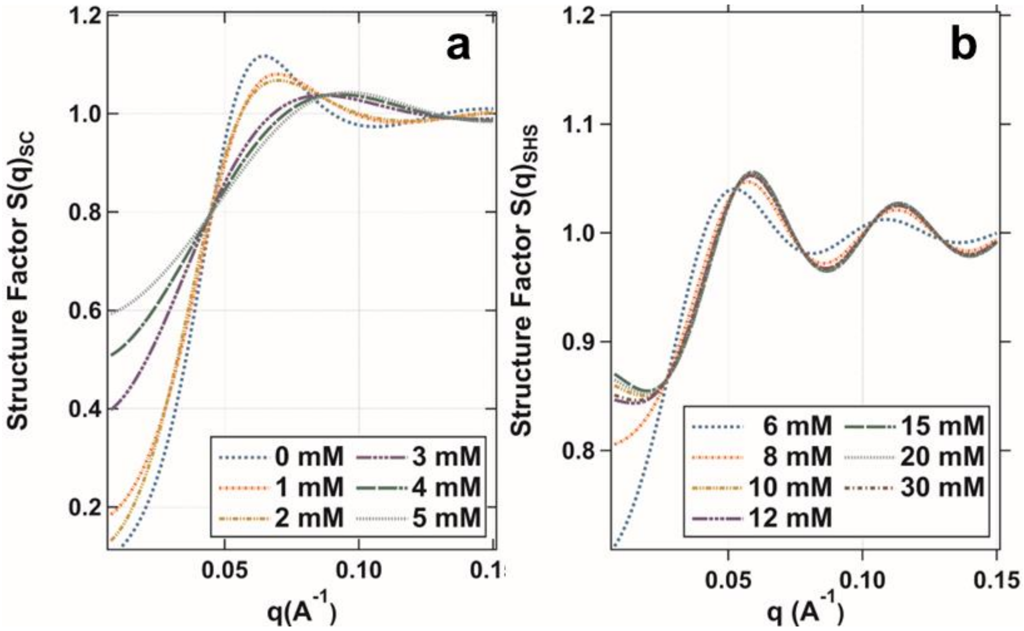


Figure S7: Structure factors calculated from Figure S2 as a function of salt concentration. (a and b) show the structure factors for 80 mg/ml HSA. Note that the shown structure factors were calculated using different potentials. Thus, the SC potential was used for figure (a), while an ESHS potential was used for figure (b). In (a), all conditions were approximated using a particle size of 68.06 Å, in (b) all conditions were approximated using a particle size of 68.06 Å.

## References


1. J. Als-Nielsen, Des McMorrow, *Elements of modern X-ray physics,* 2nd edn. (Wiley, Hoboken, 2011)
2. P. Lindner, T. Zemb (eds.), *Neutrons, X-rays, and light,* 1st edn. (Elsevier, Amsterdam [etc.], 2002)
3. J.B. Hayter, J. Penfold, Colloid & Polymer Sci (1983) doi:10.1007/BF01421709
4. F. Zhang, M.W.A. Skoda, R.M.J. Jacobs, R.A. Martin, C.M. Martin, F. Schreiber, The journal of physical chemistry. B (2007) doi:10.1021/jp0649955
5. L. Ianeselli, F. Zhang, M.W.A. Skoda, R.M.J. Jacobs, R.A. Martin, S. Callow, S. Prévost, F. Schreiber, The journal of physical chemistry. B (2010) doi:10.1021/jp9112156
6. O. Glatter, O. Kratky, *Small Angle X-ray Scattering,* 2nd edn. (Academic Press, London, New York, 1982)
7. NIST Center for Neutron Research (2010)
8. Gerhard Nägele, *The physics of colloidal soft matter* (AMAS Lecture Notes, Warsaw, Poland, 2004)
9. S.R. Kline, J Appl Crystallogr (2006) doi:10.1107/S0021889806035059
10. J.-P. Hansen, J.B. Hayter, Molecular Physics (1982) doi:10.1080/00268978200101471
11. J.B. Hayter, J. Penfold, Molecular Physics (1981) doi:10.1080/00268978100100091
12. Y. Liu, W.-R. Chen, S.-H. Chen, The Journal of chemical physics (2005) doi:10.1063/1.1830433
13. M.K. Braun, M. Grimaldo, F. Roosen-Runge, I. Hoffmann, O. Czakkel, M. Sztucki, F. Zhang, F. Schreiber, T. Seydel, The journal of physical chemistry letters (2017) doi:10.1021/acs.jpclett.7b00658
14. M.K. Braun, A. Sauter, O. Matsarskaia, M. Wolf, F. Roosen-Runge, M. Sztucki, R. Roth, F. Zhang, F. Schreiber, The journal of physical chemistry. B (2018) doi:10.1021/acs.jpcb.8b10268
